# Distinct Pools of cdc25C Are Phosphorylated on Specific TP Sites and Differentially Localized in Human Mitotic Cells

**DOI:** 10.1371/journal.pone.0011798

**Published:** 2010-07-26

**Authors:** Celine Franckhauser, Daria Mamaeva, Lisa Heron-Milhavet, Anne Fernandez, Ned J. C. Lamb

**Affiliations:** Cell Biology Unit, Institute de Genetique Humain, CNRS-UPR1142, Montpellier, France; University of Birmingham, United Kingdom

## Abstract

**Background:**

The dual specificity phosphatase cdc25C was the first human cdc25 family member found to be essential in the activation of cdk1/cyclin B1 that takes place at the entry into mitosis. Human cdc25C is phosphorylated on Proline-dependent SP and TP sites when it becomes active at mitosis and the prevalent model is that this phosphorylation/activation of cdc25C would be part of an amplification loop with cdk1/cyclin B1.

**Methodology/Principal Findings:**

Using highly specific antibodies directed against cdc25C phospho-epitopes, pT67 and pT130, we show here that these two phospho-forms of cdc25C represent distinct pools with differential localization during human mitosis. Phosphorylation on T67 occurs from prophase and the cdc25C-pT67 phospho-isoform closely localizes with condensed chromosomes throughout mitosis. The phospho-T130 form of cdc25C arises in late G2 and associates predominantly with centrosomes from prophase to anaphase B where it colocalizes with Plk1. As shown by immunoprecipitation of each isoform, these two phospho-forms are not simultaneously phosphorylated on the other mitotic TP sites or associated with one another. Phospho-T67 cdc25C co-precipitates with MPM2-reactive proteins while pT130-cdc25C is associated with Plk1. Interaction and colocalization of phosphoT130-cdc25C with Plk1 demonstrate in living cells, that the sequence around pT130 acts as a true Polo Box Domain (PBD) binding site as previously identified from in vitro peptide screening studies. Overexpression of non-phosphorylatable alanine mutant forms for each isoform, but not wild type cdc25C, strongly impairs mitotic progression showing the functional requirement for each site-specific phosphorylation of cdc25C at mitosis.

**Conclusions/Significance:**

These results show for the first time that in human mitosis, distinct phospho-isoforms of cdc25C exist with different localizations and interacting partners, thus implying that the long-standing model of a cdc25C/cdk1 multi-site auto amplification loop is implausible.

## Introduction 

Mammalian cell mitosis is a dynamic process involving inactivation of interphase signaling pathways and activation of mitotic kinases, particularly cyclin dependent kinases (cdks) [reviews 1], [ 2]. Amongst mitotic cdks, cdk1 and 2 are the most closely investigated, with cdk2-cyclin A2 functioning in late G2 and prophase and cdk1/cyclin B1 modulating progression to metaphase [reviewed 3]. Activation of cdk1-cyclin B1 involves various events including phosphorylation of threonine 161 (T161) on cdk1 [4], [ reviewed 1]; nuclear localization of cyclin B1 [reviewed 5], phosphorylation of cyclin B1 [6] and dephosphorylation of two negative regulatory sites T14 and Y15 on cdk1 [reviewed 1]. Dual specificity phosphatases required for the latter, are members of the cdc25 family [reviewed 7]. Humans have three different cdc25 proteins, cdc25A, B and C and all have been implicated in mitotic activation [8]–[11, respectively]. Human cdc25C was the first phosphatase identified in dephosphorylation of cdk1 [12] and is itself modulated by phosphorylation [13]–[17] and localization [reviewed 18]. Human cdc25C is also subject to alternative splicing in some cell types [19], [20]. At G2/M, cdc25C localizes in the nucleus in coordination with cyclin B1 [21] and at the centrosomes [22] and undergoes dephosphorylation of negative regulatory sites including serine 216 [23] and phosphorylation on at least 6 residues in human cdc25C, S/TP sites threonines (T) 48, 67, 130 and serine (S) 214 [17] and Plk1 sites S191 and S198 [24]. In transformed Hela cells, phosphorylation of S214 and S216 has been implicated in a ying/yang mechanism with phosphorylation of S214 bringing about the timely entry into mitosis [25]. Phosphorylation of S198 by Plk1 promotes cdc25C nuclear localization [21] and a proteomic screen of phospho-peptides interacting with the Plk1 binding domain identified a S-phosho-TP motif present in cdc25C at T130 as a polo-box-domain (PDB) binding motif [26], [ reviewed 27]. In Xenopus, phosphorylation of residues (T48, T67, T138 and S 287) changes cdc25C activity and is required for normal mitosis [14], [15]. Also in Xenopus, phosphorylation of T138 is linked to an interaction with PR56-delta subunit of phosphatase 2A (PP2A) [28] and may be involved in mitotic exit [29]. At least one report has suggested that T48 is phosphorylated by MAP kinase ERK2 [30]. In humans, residues T48, T67, T130 and S214 are in TP/SP motifs susceptible to phosphorylation by cdks, mitogen activated kinases, CAM kinase II and GSK3 [31], [30], [32]. In human cdc25C, we showed these sites could be phosphorylated by purified cdk1/cyclin B in vitro and were phosphorylated in vivo in mitotic cells [17]. Phosphorylation of cdc25C on SP/TP motifs is associated with cis-trans proline isomerization by PIN1 necessary for phosphatase activation [reviewed 33], [ 34], [ 35]. Incubation of human cdc25C with Xenopus mitotic extracts also increased phosphatase activity leading to a model where an auto-amplification loop involving cdc25C and cdk1/cyclin B may be implicit in mitotic activation [16].

Here, we have investigated the site-specific phosphorylation of cdc25C on proline directed threonines 67 and 130, and the cytolocalization and timing of these phosphorylations in dividing human cells. Using site-specific affinity purified antibodies for these sites, we show they are phosphorylated in mitotic cells but in distinct pools of cdc25C with different cytolocalization.

## Results

Anti-phospho-site antibodies are specific for single TP phosphorylation events.

**Figure 1:**
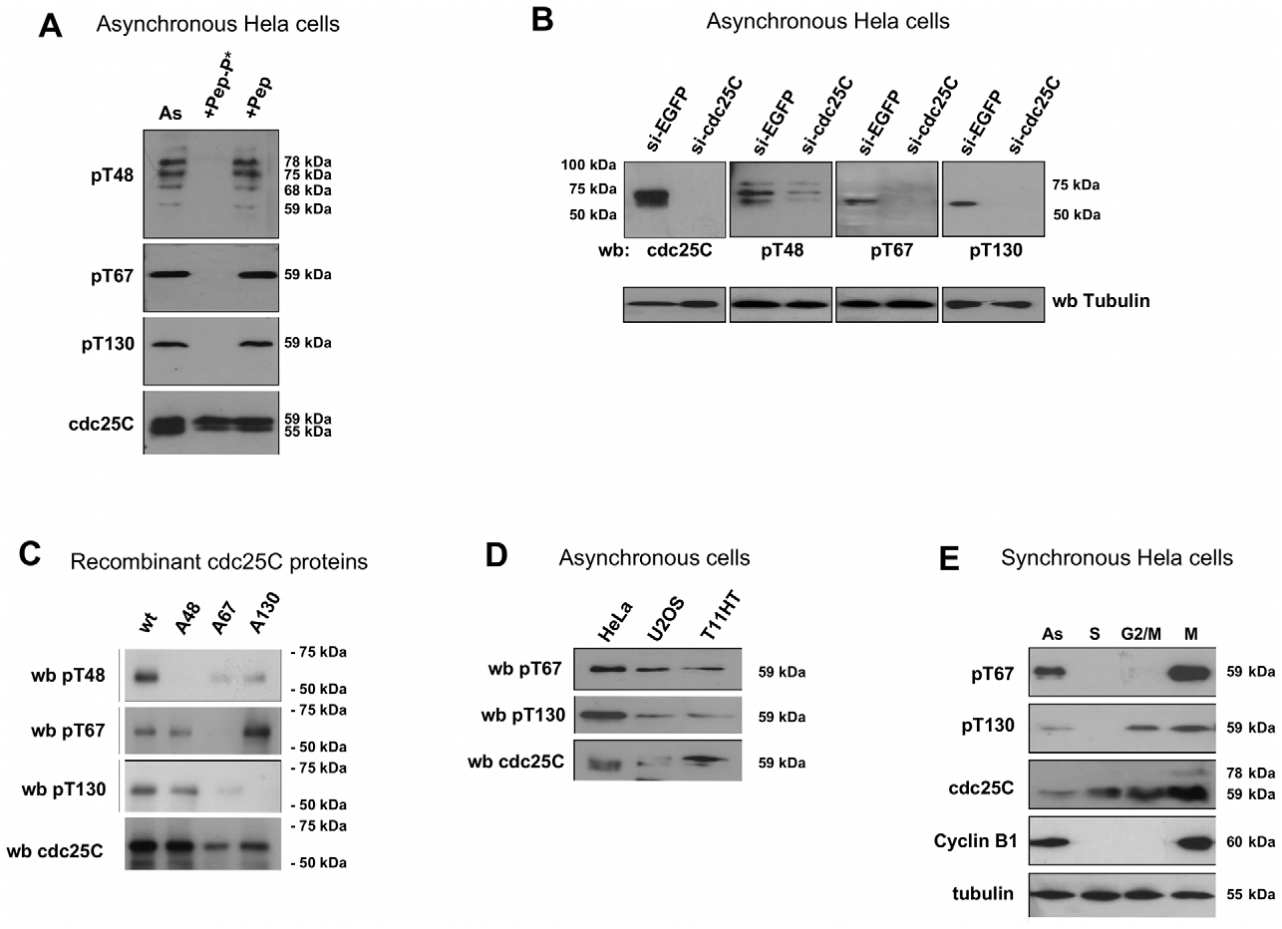
Phospho-cdc25C antibodies are specific for each cdc25C phospho epitope. Panel A: Extracts from asynchronous Hela cells were analyzed by western blotting using double affinity purified anti-phospho-cdc25C antibodies. Shown are typical protein patterns recognized by anti-pT48 (pT48), anti-pT67 (pT67) and anti-pT130 (pT130) in extracts from asynchronous cells (As) and the same extracts after the antibodies were pre-incubated for 1 hour in 50 ug/ml of the non-phosphorylated peptide epitope (+Pep) or the phosphorylated peptide epitope (+Pep-P*). Molecular masses are in kDa. Panel B: RNA interference specifically ablates the signal for pT67 and pT130 epitopes: Hela cells were transfected with siRNA targeting cdc25C and 24 hours after harvested and analyzed for the expression of cdc25C (H150) and signals for pT48, pT67 and pT130. Shown are typical western blot patterns. Also shown are loading controls (anti-alpha-tubulin). Panel C. Anti-phospho-cdc25C antibodies are specific for single phosphorylation sites on cdc25C: cdc25C Wt and alanine mutant proteins were expressed in bacteria and after purification phosphorylated in vitro using purified cdk1/cyclin B1. Shown are the western blots for WT, T48A, T67A and T130A cdc25C blotted with anti-cdc25C (H150, pT48, pT67 and pT130). Panel D. Phospho-cdc25C isoforms are present in human cells of different types. Extracts were prepared from transformed U2OS cells or normal human fibroblasts (T11-HT) and analyzed for phosphorylation of cdc25C. Shown are typical western blot of asynchronous cells profiles stained for anti-pT67, anti-pT130 and anti-cdc25C (T150). 50 ug total protein extract/lane. Panel E. Limited cell cycle analysis of the phosphorylation patterns for cdc25C in Hela cells. Cells were asynchronous (as), at S-phase (TT), late G2 (G2/M) or mitosis (M). Shown are western blots for pT67, pT130, cdc25C (H150), cyclin B1 and tubulin.

Human cdc25C is phosphorylated on three TP motifs (T48, T67 and T130) and one SP motif (S214) during mitosis in human cells [17]. In order to analyze the potential role for phosphorylation of the N-terminal of cdc25C during human mitosis we developed antibodies specific for the three phospho-TP motifs using 9 amino acid peptides as shown in Supplemental Figure S1A. After double affinity purification each antibody was tested by western blotting. To demonstrate that the antibodies were specific for the phosphorylated peptide, we incubated the affinity purified antibodies with the phospho-peptide antigen or the non phosphorylated peptide before blotting. As shown in Figure 1A, only the phospho-peptide antigen specifically blocks reactivity for each antibody by western blot against Hela cell extracts. Anti-pT48 reacts with a number of protein bands from 59–80 kDa whereas anti-pT67 and anti-pT130 react with discrete bands around 59kDa which in the case of pT67 may appear as a doublet. We also obtained the same results when the non-phospho-peptide antigen was accompanied by phospho-threonine and/or the two other phospho-peptides (pT67, pT130 for anti-pT48, pT48 and pT130 for anti-pT67 and pT48 and pT67 for anti-pT130) effectively excluding the possibility that our antibodies react with anti-phospho-T or phospho-T-proline motifs (data not shown).

To confirm that these phospho-site directed antibodies were specific for cdc25C, we next compared the protein patterns between normal asynchronous Hela extracts and extracts from Hela cells in which cdc25C had been ablated by RNA interference [36]. As shown in Figure 1B, anti-pT67 and anti pT130 recognize discrete bands similar to those shown in Figure 1 in cells transfected with non-specific siRNA duplexes and these bands are absent in extracts from cells transfected with siRNA to cdc25C. In contrast, the anti-pT48 reacts with 3 proteins of molecular masses ranging from 85 to 50 kDa only two of which are effectively reduced by siRNA to cdc25C. The same results were obtained with 3 different RNA duplexes [36], [37] used either alone or in combination. Furthermore, these same non specific bands were observed in U2OS cells and are unlikely to be spliced forms since, they also exist in non-transformed cells and the Lindquist siRNA is directed against a sequence present in all 5 spliced forms described to date. They are also present in the commercially available anti-pT48 (data not shown). Perhaps explaining the persistence of these bands after siRNA-cdc25C treatment, a motif search for the phospho-epitope peptide antigen for pT48 against the primate protein database, retrieved an entirely conserved sequence in the human inositol bi-phosphatase kinase 5 YVLE (PIP3K5), of which 4 forms 90, 75, 60 and 50 kDa are present in Genbank primate (N. lamb and A. Fernandez unpublished observation).

We next confirmed the specificity for each phospho-site on cdc25C by reacting the antibodies with Wt and alanine mutant cdc25C proteins phosphorylated in vitro by cdk1/cyclin B1. As shown in Figure 1C, anti-pT67 reacts with phosphorylated WT, T48A and T130A cdc25C but does not react with T67A mutant cdc25C. Similarly, anti-pT130 reacts with phosphorylated WT, T48A, T67A but not T130A cdc25C. The phosho-specific antibodies do not react with cdc25C unless it has been phosphorylated (Supplemental Figure S1B). The apparent differences in anti-phospho-antibody reactivity to the in vitro phosphorylated proteins shown in Figure 1C do not represent a differential sensitivity of the antibodies for the different alanine mutants but more likely the variable stability of the alanine mutant proteins. As shown in Figure S1C, when overall levels of anti-phospho-antibody staining are normalized to the levels of cdc25 present in the samples, the levels of phosphorylation of the Wt and mutant proteins are very similar. Moreover, the reactivity of the antibodies with phospho-cdc25C is completely abolished if cdc25C is briefly incubated with alkaline phosphatase for 5 minutes (data not shown). From these data we can conclude that anti-pT67-cdc25C and anti-pT130-cdc25C antibodies react exclusively and specifically with single phosphorylated epitopes on human cdc25C.

To confirm the existence of cdc25C phospho forms in other cells types, we analyzed extracts from asynchronous transformed U2OS and normal non-transformed human fibroblast. As shown in Figure 1, panel D, both pT67 and pT130 have similar blotting patterns in extracts of U2OS and non transformed fibroblast cells as those observed in Hela.

Finally, we analyzed the profile for each phospho-site anti-cdc25C in synchronized Hela cells. Figure 1E, shows that while the phosphorylation of pT67 and pT130-cdc25C is present in asynchronous cell extracts, it is absent from extracts of cells blocked in S-phase. The signal for pT67 is not present in late G2 cells (TT+8) but strongly stains extracts from mitotic cells. Anti-pT130 also strongly reacts with asynchronous and mitotic extracts and not S-phase blocked cells. However, unlike pT67 antibodies, pT130 also reacts with cdc25C in extracts from late G2 (TT+8) suggesting that this site is phosphorylated earlier in mitosis. These data demonstrate the specificity of these two antibodies for single phospho-epitopes on human cdc25C.

### Different cytolocalization for anti-phospho-cdc25C pT67 And pT130

**Figure 2:**
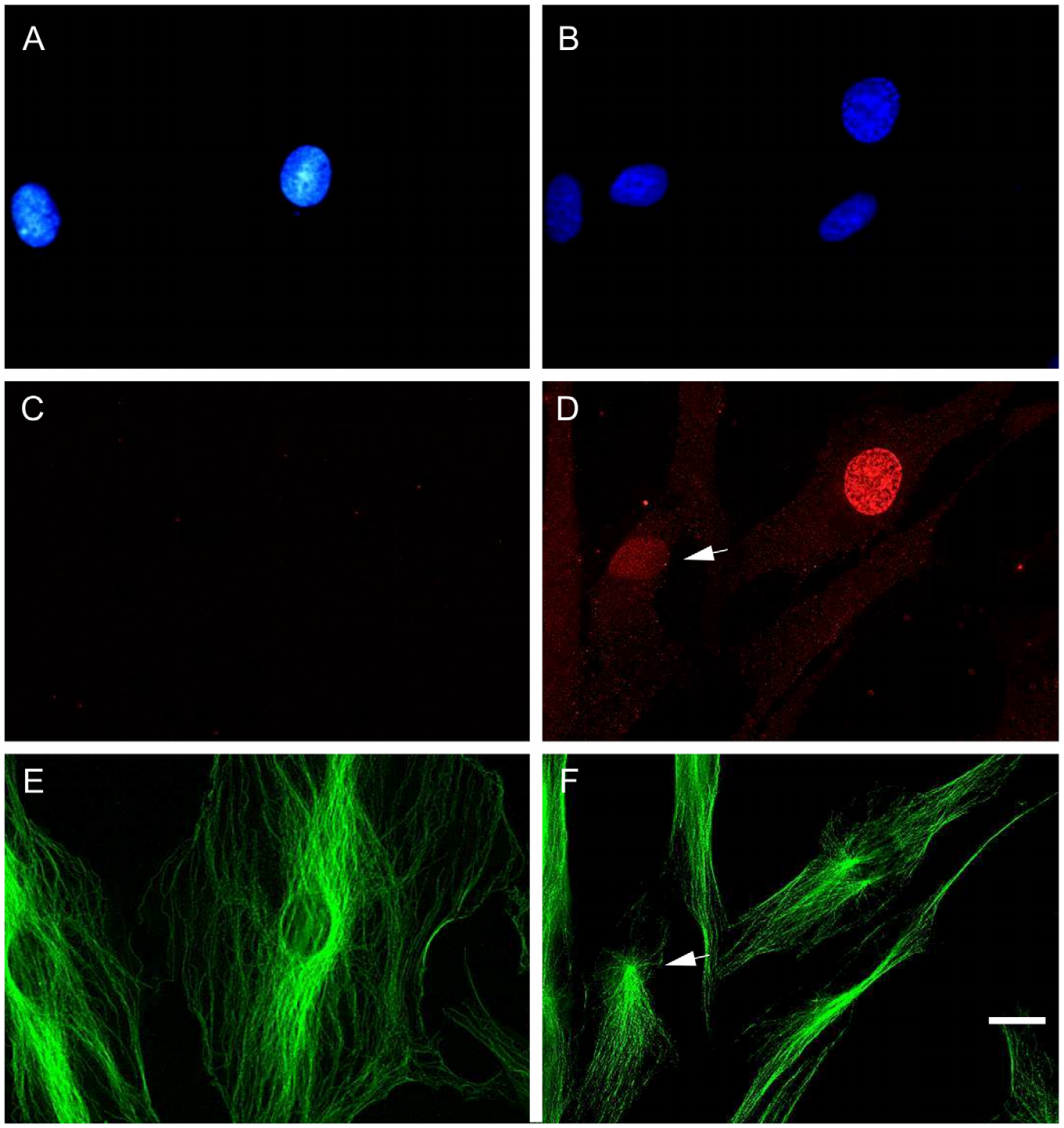
Cdc25C is phosphorylated on threonine67 only in mitosis and localizes in the nucleus in prophase. Human fibroblasts were synchronized by serum deprivation and fixed in G1/S (16 hours after refeeding) and at G2/M (26 hours post refeed). Cells were stained for DNA (A, B), anti-cdc25C-pT67 (C,D) and tubulin (E,F). Shown are representative fluorescence photo micrographs of staining in G1/S phase cells (panels A, C, E) and G2/M phase cells (panels B, D, F). Phospho-pT67 staining is absent from G1 cells but present in the nucleus of late G2 (arrowed in D) or prophase cells. Bar 10 uM.

**Figure 3:**
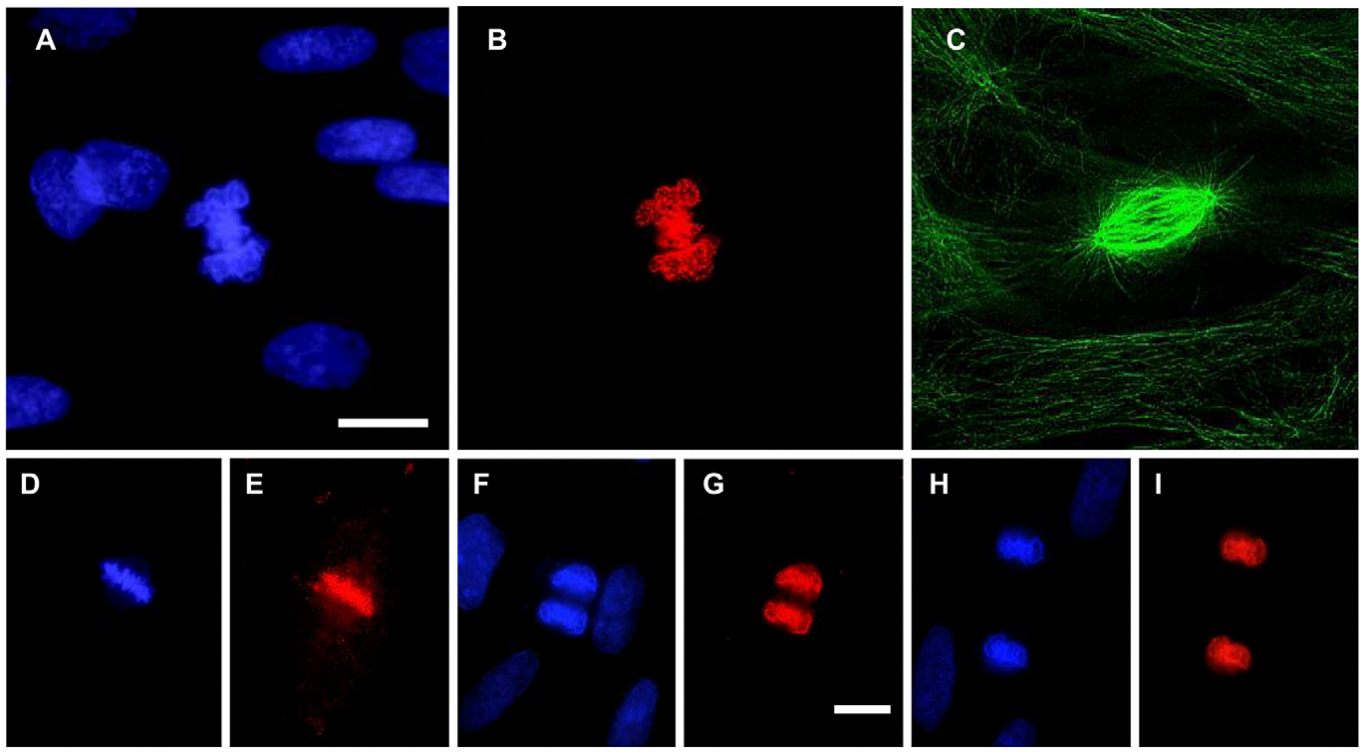
Threonine 67 cdc25C is phosphorylated during mitosis and associates with the condensed chromatin. Asynchronous normal human fibroblasts were fixed and stained for DNA (panels A, D, F, H), anti-cdc25C-pT67 (panels B, E, G, I) and tubulin (panel C). Shown are fluorescence photo micrographs of typical staining patters in cells in prometaphase (A–C), metaphase (D–E), anaphase A (F–G) and anaphase B (H–I). Bar 5 uM.

**Figure 4:**
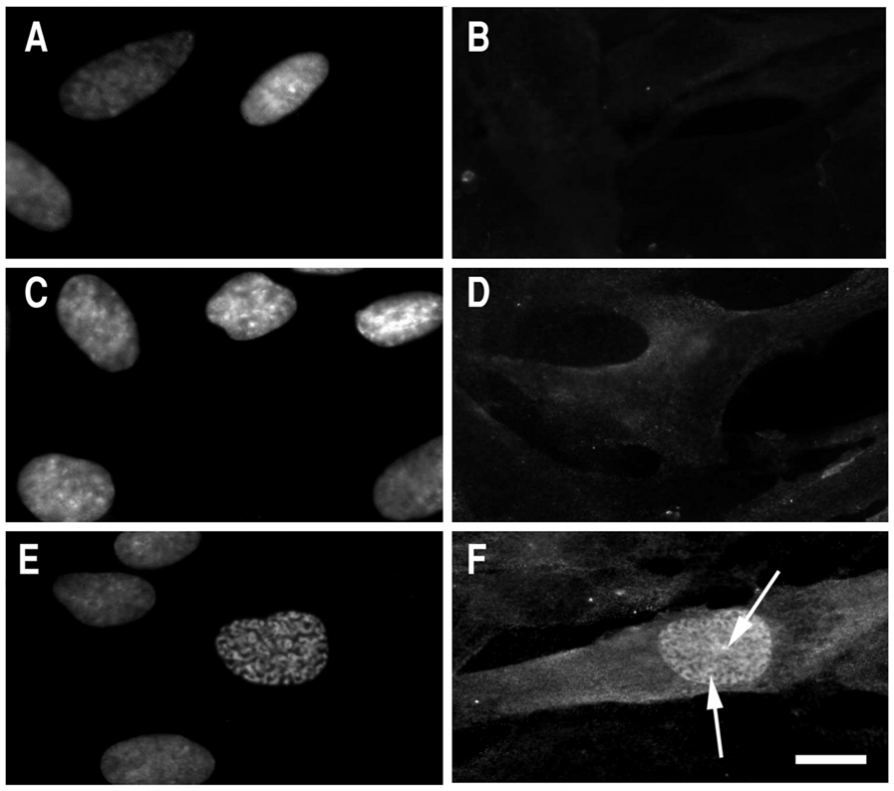
Human cdc25C is phosphorylated on threonine 130 only in mitosis. Normal human fibroblasts were synchronized by serum deprivation and fixed in G1 (12 hours after refeeding), S-phase (17 hours after refeeding) and at G2/M (26 hours after refeeding). Cells were stained for DNA (A,C,E) and anti-pT130-cdc25C (B,D,F). Shown are fluorescence photo micrographs of typical staining patterns of cells in G1 (panels A–B), S-phase (panels C–D) and G2/M (panels E–F). Staining for pT130-cdc25C is absent from G1 and S-phase cells (panels B and D) but present in the nucleus of prophase cells (panel F). Arrowed are the positions of the two centrosomes in panel F. Bar 5 uM.

**Figure 5:**
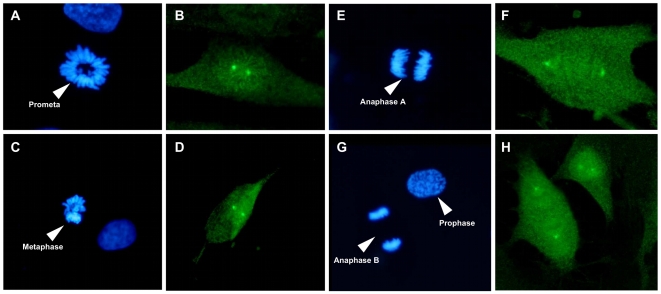
Human cdc25C phosphorylated on threonine 130 localizes to the centrosome during mitosis. Asynchronous normal human fibroblasts were fixed and stained for DNA (panels A, C, E, G), anti-cdc25C-pT130 (panels B, D, F, H). Shown are fluorescence photo micrographs of typical staining patterns of cells in prometaphase, (A–B), metaphase(C–D), anaphase A (E–F) and anaphase B (G–H). An early prophase cell is also visible in panels G and H). Bar 5 uM.

We next analyzed the cytolocalization of anti-pT67 and anti-pT130 in synchronized human fibroblasts (the same cells as blotted in Figure 1D). As shown in Figure 2, anti-pT67 does not react with cells in G1/S-phase and only becomes visible in cells in late G2 (Figure 2, arrowed) or entering mitosis (prophase) where it localizes in the nuclear compartment. The signal in the mitotic cell is specific for pT67 since pre-incubation of the antibodies with phospho-peptide (but not non-phosphorylated peptide) before use completely abolishes the signal in the mitotic cells (Figure S2). More detailed analysis of mitotic cells (Figure 3), shows that pT67-cdc25C is closely associated with the chromatin from prophase until the end of telophase. Furthermore, a similar chromatin association is seen in other human cell types such as U2OS (Figure S3). In a manner similar to anti-pT67, anti-pT130-cdc25C does not detect any signal in G1 or S-phase cells (Figure 4) or in G2 cells until the entry into mitosis. At this time, in contrast to anti-pT67, in addition to the general increase in pT130 staining, anti-pT130 localizes at two distinct points reminiscent of the centrosomes (arrowed Figure 4F). In a manner similar to anti-pT67, the signal for pT130 is specifically abolished by pre-incubation of the antibodies with the phospho-peptide antigen but not the non-phospho-peptide antigen (Figure S4) and is found in other cell types such as U2OS (Figure S4, E and F) or Hela (data not shown). More detailed analysis during mitosis (Figure 5), confirms that pT130-cdc25C localizes primarily at the spindle poles throughout mitosis until the end of anaphase. Staining normal human cells for endogenous cdc25C (Figure S5) reveals that both nuclear and spindle pole cytolocalization for phospho-cdc25C are detected in normal mitotic cells. We have also microinjected siRNA duplexes to cdc25C into human fibroblasts and confirmed that the signal for both phospho-forms of cdc25C was abolished by cdc25C knock-down, although since siRNA injected cells are blocked before entering mitosis, this result is to be expected (data not shown). These data further show that cdc25C molecules are specifically phosphorylated on these two sites only at mitosis and for the first time that these phospho-isoforms of cdc25C have specific spatially distinct localizations in mitotic cells.

### Human cdc25C is mono-phosphorylated on TP/SP motifs at mitosis

**Figure 6:**
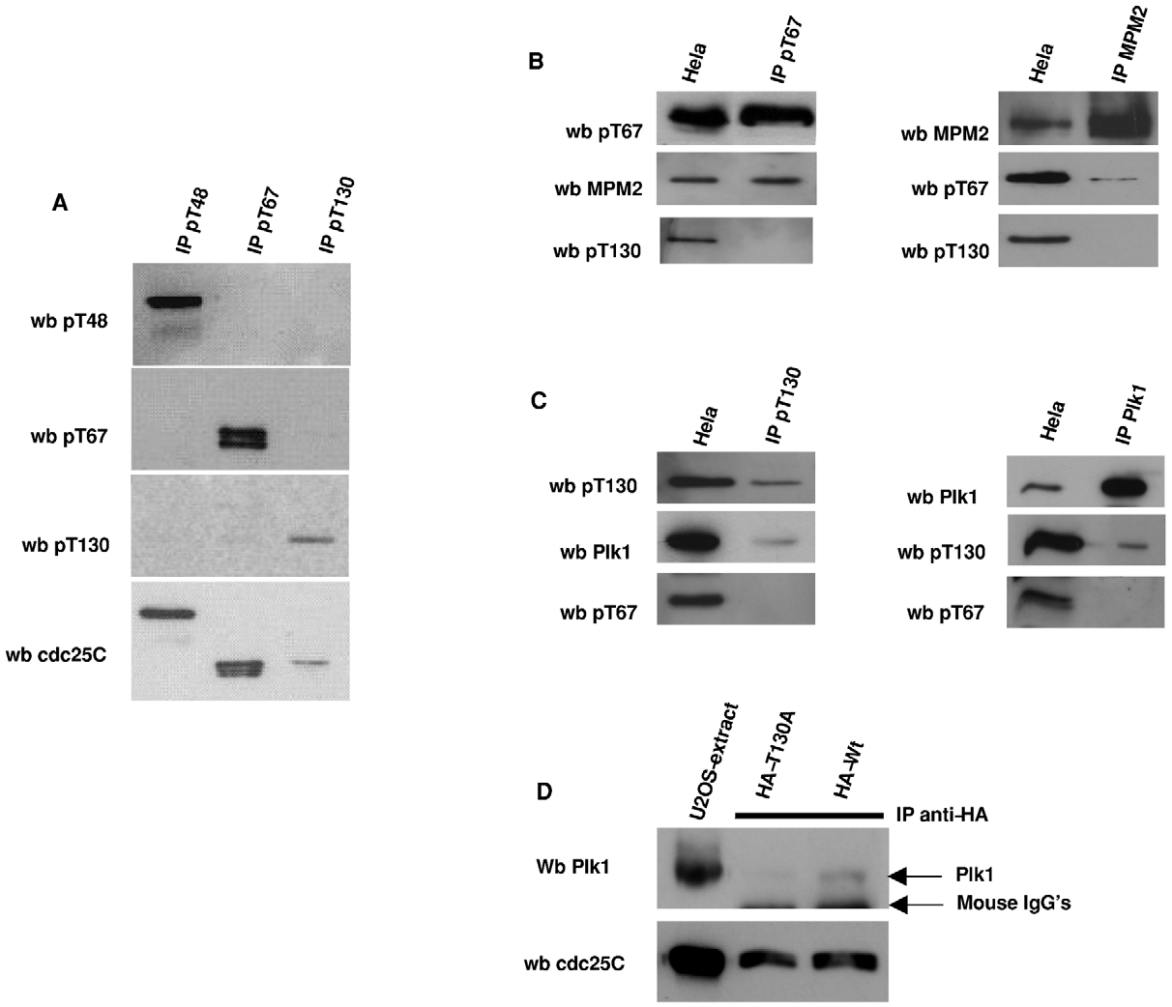
Phospho-cdc25C isoforms are not phosphorylated on other TP sites and associate with different proteins. Panel A: Phospho-cdc25C isoforms are exclusively phosphorylated on a single TP motifs at any one time. Phospho-cdc25C isoforms were immuno-precipitated from Hela cells using anti-phospho-cdc25 antibodies directed against pT48, pT67 and pT130 and blotted for each phospho-isoform and anti-cdc25C. Immunoprecipitates were separated by PAGE before transfer to nitrocellulose. Panels B and C; Extracts from mitotically enriched HeLa cells were immunoprecipitated with anti phospho-cdc25C pT67 or pT130 before subsequently probing for associated proteins. Panel B shows a typical membrane probed for pT67, MPM2 and pT130-cdc25C (left panel). Right panel shows the converse experiment in which MPM2 proteins were immunoprecipitated and then probed for anti-MPM2, anti-pT67 or anti-pT130. Panel C shows a similar experiment immunoprecipitating anti-pT130-cdc25C and probing for pT130-cdc25C, anti-Plk1 and anti-pT67 (left panel). Right hand panel shows the converse experiment immunoprecipitating anti-Plk1 and probing for anti-Plk1, anti-pT130-cdc25C and anti-pT67-cdc25C. Panel D, HA-tagged cdc25C Wt and cdc25C-T130A were transfected into Hela cells and allowed to express for 24 hours. HA-tagged proteins were immunoprecipitated from cells extracts in the presence of protease and phosphatase inhibitors before PAGE and transfer. Membranes were probed for cdc25C expression (anti-C20) and the Plk1. Shown are an extract from untransfected U2OS, immunoprecipitated HA-cdc25C-T130A (middle lane) and HA-cdc25C-Wt (right hand lane). Arrowed are the mouse heavy chains and the position of Plk1.

The discovery that different phospho-cdc25C isoforms have distinct localizations lead us to question whether cdc25C was uniquely phosphorylated on the different TP sites. We immunoprecipitated each endogenous phospho-cdc25C isoforms from HeLa cells with anti-pT48, pT67 and pT130 and after separation by western blot, re-probed the three precipitates with each phospho-specific antibody or anti-cdc25C. Throughout the immunoprecipitation process, cell extracts were incubated with antibodies in the presence of phosphatase inhibitors (Okadaic acid, Tautomycin and calyculin A) and phosphatase attenuators, PBS, beta-glycero-phosphate, sodium vanadate and fluoride (c.f. Materials and methods). As shown in Figure 6A, each phospho-isoform reacts only with itself and not with any proteins precipitated by the other three antibodies. In addition, the proteins immunoprecipitated by each of the anti-phospho-antibodies and recognized by these antibodies were also recognized by anti-cdc25C C20. The lack of additional bands previously detected by immunoblot with anti-pT48 (Fig 1A) is likely due to the clearing centrifugation step which precedes immunoprecipitation removing the cell membranes and associated membrane bound PIP3K5 in the insoluble fraction. These data show for the first time that the 3 TP mitotic phosphorylation sites in human cdc25C are only phosphorylated on a single TP motif and not simultaneously phosphorylated on the other two TP motifs.

**Figure 7:**
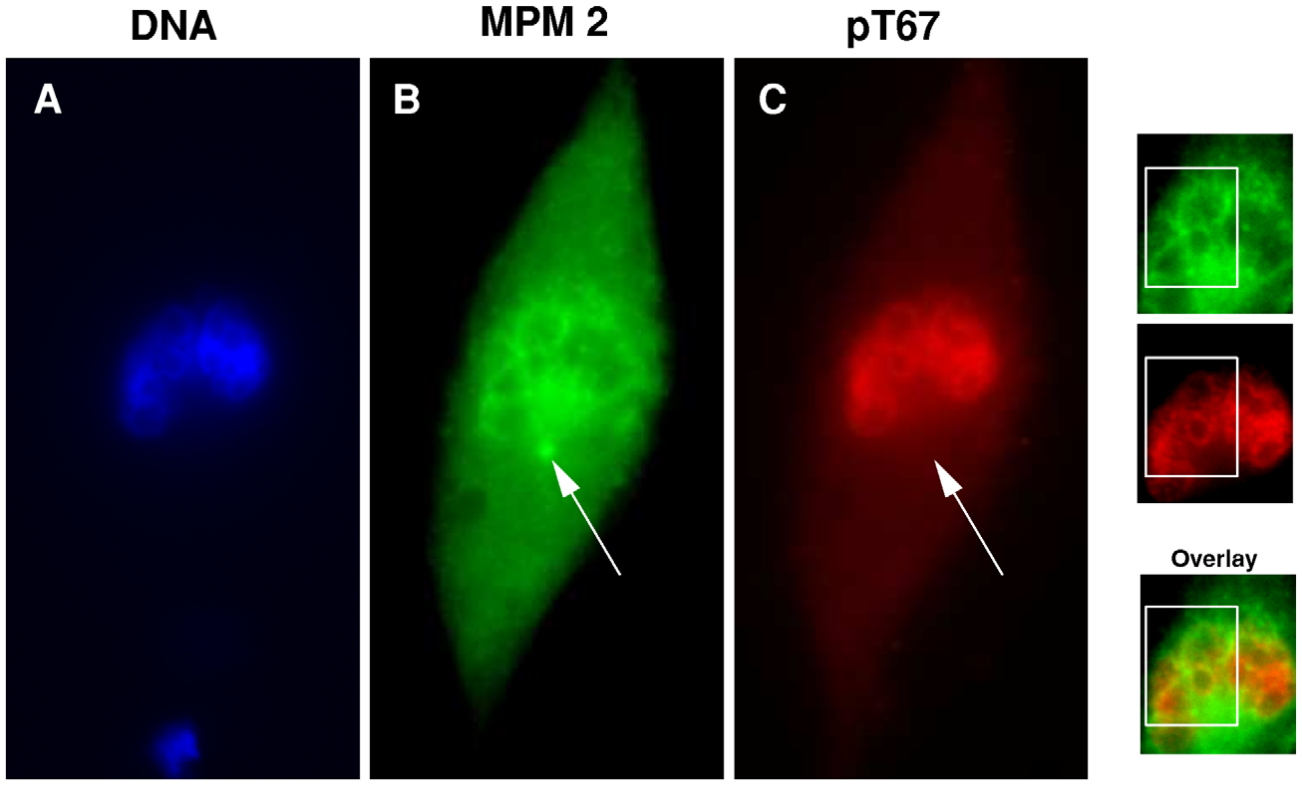
Immunofluorescence staining for anti-pT67 and MPM2 partially overlap around the chromatin. Asynchronous human fibroblasts were probed by double immunofluorescence for MPM2 antigens and pT67-cdc25C. Shown are typical fluorescence micrographs of staining for DNA, MPM2 and pT67-cdc25C. Side panels show a limited region of each image and the images overlapped. Arrowed are the position of the centrosome stained only by anti-MPM2.

The cytolocalizations observed for pT67-cdc25C and pT130-cdc25C are similar to those described for a number of mitotic factors including MPM2 antigens [38], Plk1 and gamma-tubulin. We therefore probed the immunoprecipitates of endogenous anti-pT67 and anti-pT130 for the presence of other interacting proteins. As shown in Figure 6B and C, phospho-T67-cdc25C is associated with MPM2 reactive proteins, but not with Plk1 and this association was observed both by precipitating MPM2 or pT67-cdc25C. In contrast, pT130-cdc25C co-precipitated Plk1 but not MPM2 proteins. These co-immunoprecipitation experiments further show that anti-pT67 cdc25C is not associated with pT130 cdc25C and vice versa. The association of pT130-cdc25C with Plk1 is consistent with the localization of Plk1 [39] and homology between the phospho-T130 motif and the previously identified polo-box domain (PBD) binding motif [26], [ reviewed 27]. We therefore examined if mutation of the putative PBD motif in cdc25C interfered with the interaction with Plk1. As shown in Figure 6D when HA-tagged cdc25C expressed in U2OS cells is immunoprecipitated through the HA-tag, Plk1 is associated with the tagged protein. In contrast, when immunoprecipitates of HA-tagged cdc25C-T130A are probed for Plk1, the signal is less than 10% of that observed for the Wt despite the HA-tagged proteins being expressed at quantitatively similar levels. These data show that not only are the phospho-isoforms differently localized in mitotic cells and not simultaneously phosphorylated, but they are also associated with different protein partners. This association is also seen by indirect immunofluorescence. As shown in Figure 7, when normal fibroblasts are simultaneously stained for MPM2 antigens and pT67-cdc25C a proportion of the staining for MPM2 antigens overlaps with that of pT67-cdc25C around the condensed chromatin. However, the staining for the MPM2 antigens, which also recognizes the centrosome, does not overlap with that of anti-pT67-cdc25C (arrowed in Figure 7).

### Phospho-T130-cdc25C is localized at the centrosome

**Figure 8:**
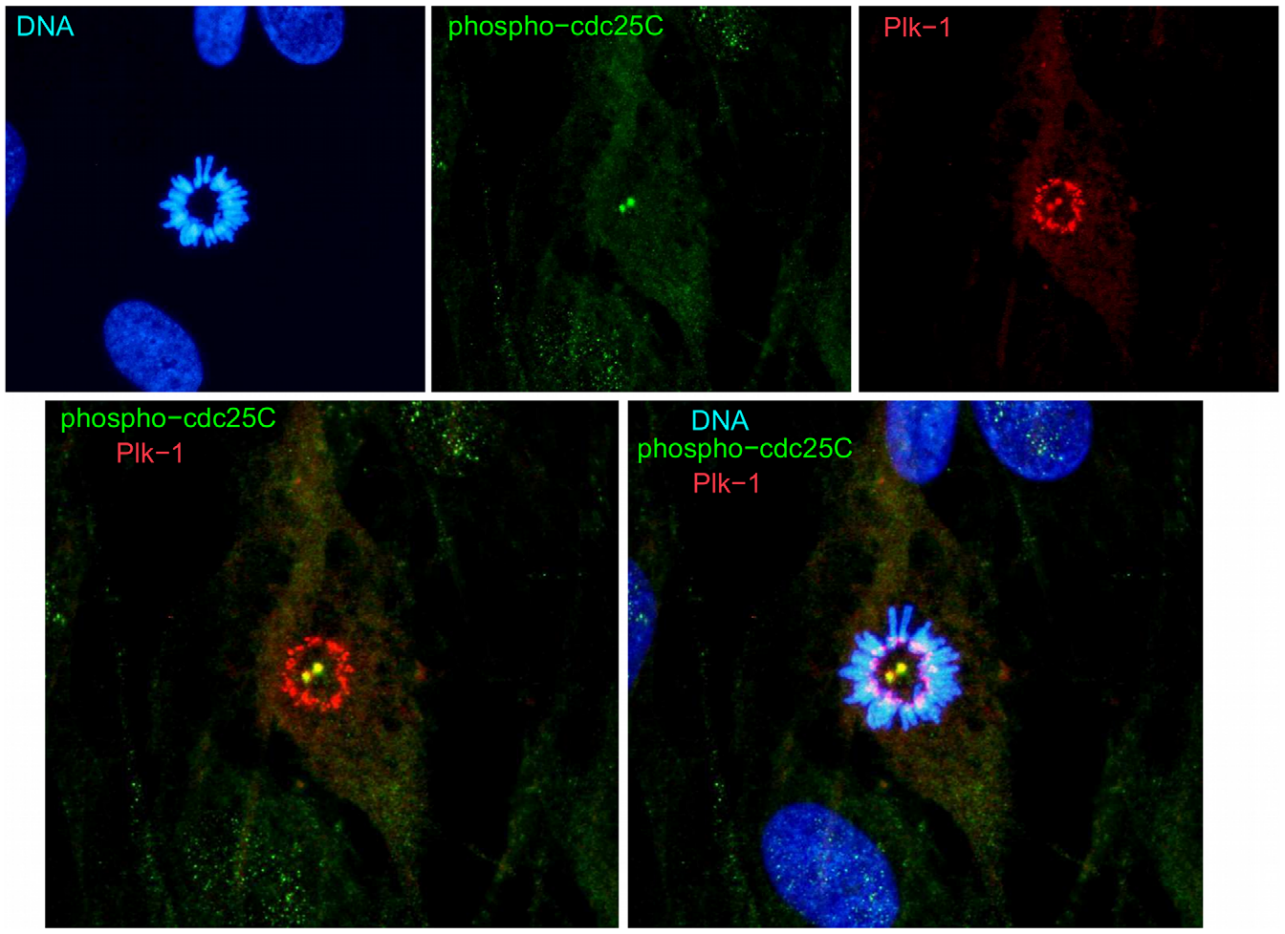
Phospho-pT130-cdc25C and Plk1 co-localize at the centrosomes but not at the centromeres. Normal human fibroblasts were stained for DNA, Plk1 and phospho-T130-cdc25C. Shown are the staining for DNA alone, pT130-cdc25C alone, Plk1 alone, pT130-cdc25C and Plk1 overlapped and all three staining patterns overlapped.

**Figure 9:**
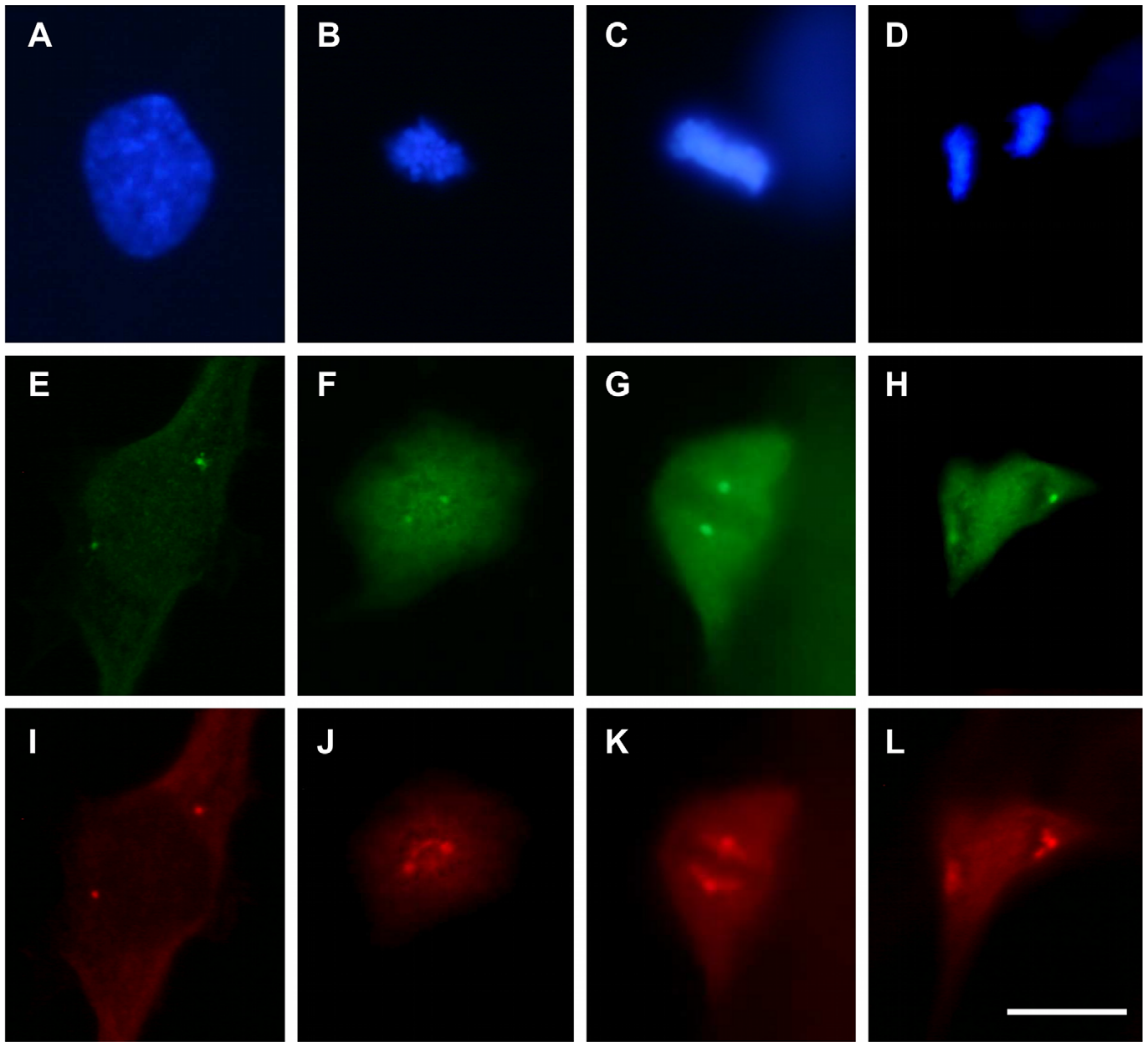
Anti-phospho-pT130-cdc25C localization coincides with staining for anti-gamma tubulin. Normal human fibroblasts were fixed and stained for DNA (panels A–D), anti-pT130 (panels E–H) and anti-gamma tubulin (panels I–L). Shown are photo micrographs of typical staining patterns in cells in prophase (panels A, C, I), prometaphase (panels B, F, J), metaphase (panels C, G, K) and anaphase (D, H, L). Bar 5 uM.

**Figure 10:**
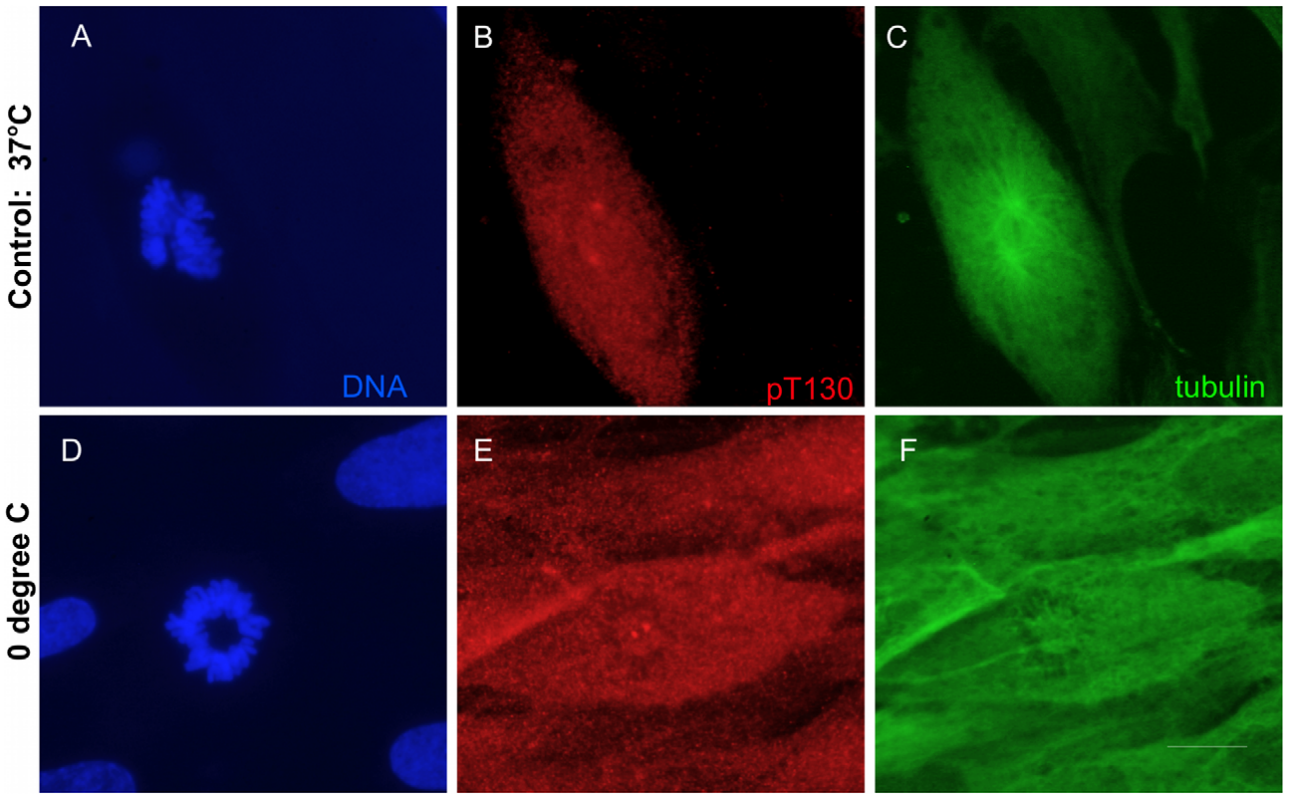
Centrosomal localization of pT130-cdc25C resists microtubule and spindle disassembly at 4°C. Normal human fibroblasts were incubated at 4°C for 60 minutes before fixation and staining for DNA (panels A, D), anti-pT130-cdc25C (panels B, E) and anti-tubulin (panels C, F). Shown are typical fluorescence photo micrographs of an untreated cell in prometaphase (panels A–C), and a polar view of a metaphase cell after 60 minutes at 4°C (panels D–F). Note the persistence of centrosomal staining in E with the absence of any microtubule spindle in F.

To confirm the centrosomal localization of cdc25C-pT130, we undertook detailed confocal analysis of Plk1 and pT130-cdc25C cytolocalization under different conditions. As shown in Figure 8, at metaphase, Plk1 and pT130-cdc25C are perfectly superimposed at the spindle poles. Interestingly, the Plk1 associated with the centromeres at metaphase (Plk1 staining of the internal ring of chromosomes) is not associated with pT130-cdc25C. To confirm that pT130 was truly present at the centrosomes, we performed co-localization of pT130-cdc25C with gamma-tubulin. As shown in Figure 9, the gamma-tubulin and pT130-cdc25C staining superimpose at the spindle poles. Moreover, (Figure 10), pT130-cdc25C remains associated with the centrosomes even when cells are chilled to 4°C for 60 minutes to disrupt the microtubule network and spindle. In contrast, under similar low temperature conditions (4°C, 60 or 120 minutes) Plk1 no longer localizes at the centrosomes even though cdc25C-pT130 staining remains robust (Supplemental Figure S6). These data confirm that pT130-cdc25C is a phospho-form of cdc25C associated with the centrosomes and spindle poles during mitosis in human cells.

**Figure 11:**
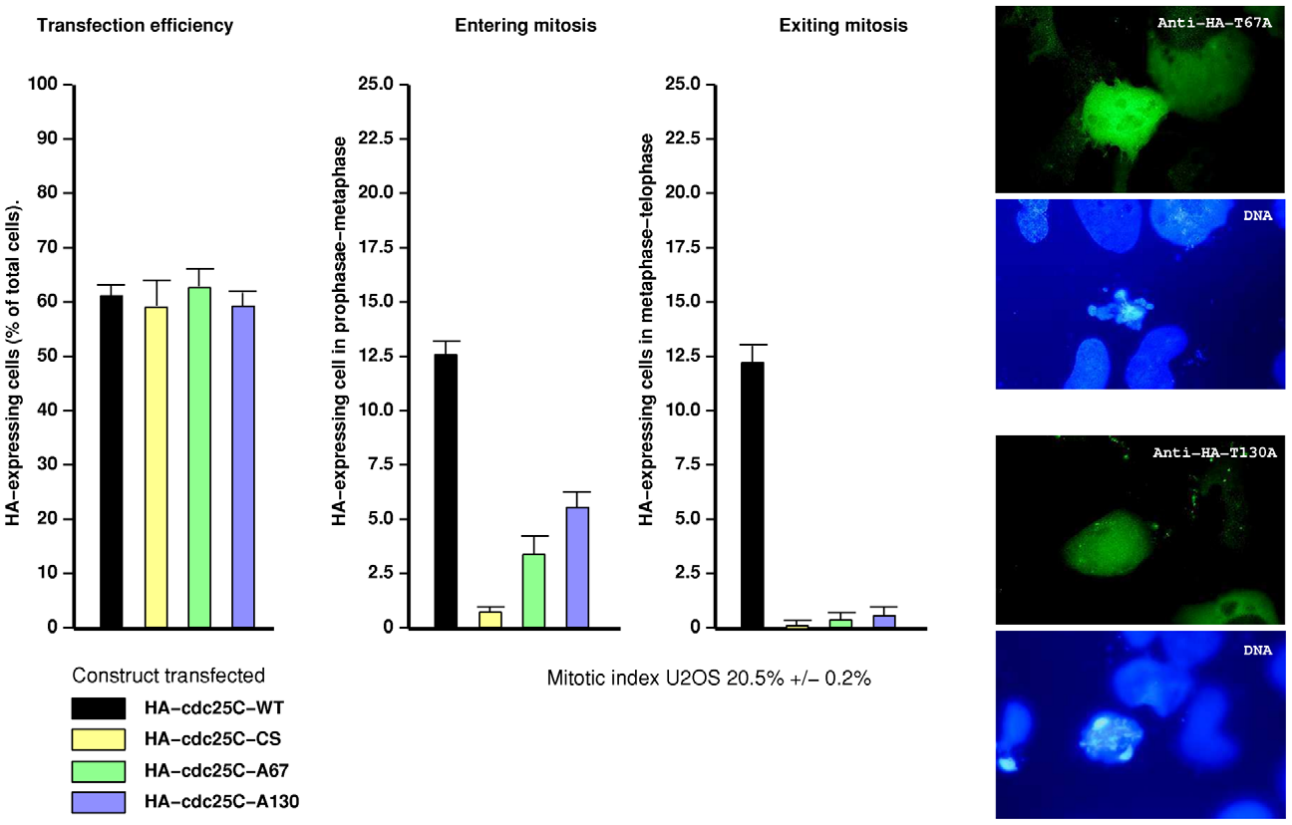
Transient transfection of non-phosphorylatable mutants of cdc25C significantly delay mitosis. Asynchronous U2OS cells were transfected with plasmids expressing HA-tagged Wt-cdc25C, catalytically inactive C377S-cdc25C, T67A-cdc25C and T130A-cdc25C. 24 hours after transfection cells were fixed and stained for Ha-cdc25C and scored for transfection efficiency (percentage of transfected cells per 100 cells) and mitotic figures. Mitotic entry: cells with condensed chromatin, centrosomal separation and spindle formation; or mitotic exit: cells with separated chromatin, anaphase spindles or mid-bodies. Shown are the results of 6 typical separate transfection experiments. Also shown are fluorescence micrographs of typical staining for anti-HA-T67A-cdc25C and HA-T130A-cdc25C expressing cells and the corresponding DNA staining.

Finally, we examined the effects of transfecting cells with non-phosphorylatable mutant forms of cdc25C into U2OS cells and scoring mitotic progression. As shown in Figure 11A, wild type cdc25C, the phosphatase inactive C377S, T67A and T130A HA-tagged cdc25C mutant forms are expressed at equal levels after transfection. We then scored the mitotic stage of the HA-expressing cells for either cells entering mitosis (prophase-metaphase) or cells exiting from mitosis (anaphase A to telophase). The overall background mitotic index is around 20%. In cells transfected with Wt-cdc25C, 10 to 12% where at some stage of mitotic entry and an equal percentage in mitotic exit (data taken from 6 different experiments). As to be expected, in cells expressing C377S-cdc25C, a phosphatase inactive dominant-negative form of cdc25, no cells were observed in mitotic exit, and less than 1% showed any form of chromatin condensation. No cells were in prometaphase or metaphase. No cells expressing either T67A-cdc25C or T130A-cdc25C were observed in mitotic exit (anaphase-telophase). Cells expressing T67A also showed a significant decrease in entering mitosis with less than 4% of cells having condensed chromatin. Cells expressing T130A-cdc25C had a 50% decrease in early mitotic figures but were generally more rounded than cells transfected with T67A. None of the cells expressing T67A or T130A were observed in metaphase. Interestingly, when cells expressing only low levels of HA-tagged cdc25C-T67A or T130A are examined, a proportion of the endogenous cdc25C proteins still localize correctly while the overexpressed alanine mutants do not (Supplemental Figure S7) suggesting that these alanine mutants function as competitive inhibitors restricting access of endogenous cdc25C to essential cellular factors. Similar inhibition rates were obtained using Hela cells or in non-transformed diploid fibroblasts (T11-HT). Taken together, these data show that mitotic phosphorylation of cdc25C on T67 and T130 perform essential functions in mitotic progression, and that pT67-cdc25C and pT130-cdc25C isoforms have different localizations during human cell mitosis, are not simultaneously phosphorylated on other TP sites and are associated with different protein partners.

## Discussion

We show here differential spatial localization of 2 phospho-isoforms of cdc25C. We have developed mono-specific antibodies to the proline-linked phosphorylation sites on human cdc25C which are phosphorylated at mitosis. The antibodies demonstrate for the first time that different phospho-forms of cdc25C exist which have differential spatial organization during human mitosis and associate with different partner proteins. Furthermore, each phosphorylated isoform of cdc25C is not simultaneously phosphorylated on the other TP sites on the same cdc25C molecule. Finally, we show that non-phosphorylatable mutant forms of cdc25C impair mitotic progression in human cells. These data are the first to identify that separate phospho-forms of cdc25C with specific localizations and protein partners exist in human cells, thus questioning the long standing model of an auto-amplification mechanism involving cdc25C and cdk1 in mitotic activation.

### Mono-specific phospho-site antibodies to human cdc25C

We have developed antibodies to the proline dependent kinase motifs T48P, T67P, T130P which we have previously identified as being phosphorylated during human mitosis [17] and two of which: T67 and T130 are highly conserved in cdc25C from different species (Fig. S1). While each of the antibodies are highly specific for the corresponding phosphorylated peptide antigen, RNA interference knockdown of cdc25C shows that antibodies against T48 react with phospho-proteins other than cdc25C. Stringent database searching with hexamer peptide sequences corresponding to the antigen sites identified protein families with hexamer sequences corresponding to antigens for pT48 and pS214. The first family, phosphatidylinositol-3- phosphate/phosphatidylinositol 5-kinases (PIKFyve), harbor the sequence PRTPVG comprising 6 of the 9 amino acids in the pT48 antigen. In Xenopus, the equivalent site on cdc25C has been proposed to be a substrate for MAP kinases [30]. The same study reported the phosphorylation of GST-human cdc25C in vitro by MAP kinase ERK2, recognition of this phospho-motif by a commercial antibody and a reduction in human cdc25C T48 phosphorylation after inhibition of MAP kinase ERK2 with UO126. In our hands, the commercial antibody directed against human pT48-cdc25C used by these authors detects the same none cdc25C proteins as our anti-pT48 in particular those not ablated by siRNA both by western blotting and immunofluorescence likely to correspond to PIKFyve family proteins (CF, NL unpublished observation). In addition, it is unlikely that MAP kinase phosphorylates T48 in human cdc25C during normal cell mitosis since active MAP kinase cannot be detected in mitotic Hela cells in the absence of drug-induced microtubule destabilization [40].

We show here that human cdc25C phosphorylated on T130 is localized at the centrosomes during mitosis in both normal and transformed cells. Furthermore pT130-cdc25C phospho form physically interacts with the mitotic kinase Plk1 which is known to phosphorylate cdc25C on serine 198 [21]. We have observed spindle pole-centrosomal localization of endogenous cdc25C in mitotic human fibroblasts (Figure S5) and ectopically expressed fluorescent human-cdc25C has been reported to localize at the centrosomes [22]. In Xenopus, phosphorylation of T138 (the equivalent site) is important for the interaction of cdc25C with phosphatase type 2A [28]. The interaction of these two endogenous proteins Plk1 and pT130-cdc25C we showed here demonstrates in living human cells that the polo box domain (PBD) binding motif previously identified from in vitro screening analysis [26] is a functional PBD binding site. Indeed, incubating lysed cell models with a peptide containing a similar motif interfered with Plk1 localization at the centrosome [26]. We have also observed that phosphorylation of cdc25C-pT130 is essential to Plk1 localization at the centrosomes since microinjection of the phospho-peptide disrupts Plk1 localization (NL, AF unpublished observations). Furthermore, the T130A mutated form of cdc25C is significantly impaired in its interaction with Plk1 as shown by the very low signal for Plk1 associated with the HA-tagged cdc25C-T130A immunoprecipitate when compared to Wt HA-tagged-cdc25C. However, the observation that anti-cdc25-pT130 can immunoprecipitate the Plk1 raises the question of how our antibody and Plk1 can be simultaneously binding to the same epitope. One likely answer is that cdc25C-pT130 and Plk1 are part of a much larger complex of proteins associated with the centrosome or that cdc25C acts as in a multimeric form. We have biochemical data to support that human cdc25C is dimeric or tetrameric in U2OS and Hela cells (NL. CF unpublished data) and the presence of signal for Plk1 (albeit relatively low) observed when HA-tagged-cdc25C-T130A is immunoprecipitated is consistent with existence of Wt-T130A complexes. The detailed biochemical characterization of phospho-cdc25C complexes is currently in progress.

The centrosomal localization of phospho-cdc25C is resistant to microtubule destabilization by cold showing true centrosomal localization of pT130-cdc25C, i.e. not dependent upon intact spindle poles. Interestingly cdc25B has also been implicated in modulation of cdk1 activity at the centrosome [41] and has recently been identified as a substrate for Plk1 [42]. The observation that phospho-pT130 form of cdc25C is the form localized to the centrosomes and acts to anchor Plk1 at this site offers the interesting possibility that cdc25C is involved in the modulation of cdc25B by Plk1. Indeed, cdc25B does not posses a potential phosphorylated motif to bind PBD as present in pT130-cdc25C.

### Non-phosphorylatable cdc25C mutants block proliferation and delay mitotic progression

We show that expression of non-phosphorylatable (T67A and T130A) forms of cdc25C in U2OS inhibit cell cycle progression at mitosis in a manner similar to the dominant negative phosphatase inactive C377S form of cdc25C (Figure 11). For T130A-cdc25C c.a 1 cell in 100 is in mitotic exit whereas the control level is 1/10 for non transfected cells or cells transfected with WT cdc25C (Figure 11). At low levels of expression, neither cdc25C-T67A nor T130A disrupt the localization of endogenous cdc25C (Figure S7), suggesting that the effects of higher levels of expression on mitosis transit are due to competitive inhibition. The implication of cdc25C in cell cycle transit, checkpoint signaling and mitotic activation has been well established over a considerable period of time [11], [9]. However, Lindquist and coworkers [37] reported only a transient delay in Hela cells microinjected with siRNA targeting cdc25C and suggested that cdc25C was not essential to mitosis in highly transformed cells. In our hands, microinjection of either the siRNA sequences described by Lindquist et al., [37] or our own [36] at 10 nM in the needle, ca∼1.0 nM in the cell together with plasmids expressing EGFP as marker was accompanied by a complete absence of mitotic cells and no staining for either pT67 or pT130 in both U2OS or normal diploid fibroblasts (data not shown). Varmeh & Manfred [43], demonstrated that overexpression of cdc25C sensitized U2OS cells to deoxyrubicin an effect not seen in non-transformed cells. Overexpression of cdc25C did however, block non transformed cells at the G1/S boundary [43]. However, these authors also reported that RNA interference for cdc25C failed to block cell cycle progression in transformed U2OS cells using liposome based transfection. As we described previously [36], one of the most difficult aspects of RNA interference for cdc25C stems from obtaining a complete ablation of the cdc25C signal. Both Lindquist et al., [37] and Varmeh & Manfredi [43] demonstrate a complete loss of cdc25A protein expression by western blot while still showing residual protein expression for cdc25C. Using calcium phosphate (as opposed to liposomal methods) we could clearly demonstrate a complete loss of both cdc25A and C expression [36]. Erikson and co-workers also recently described that differential penetration of shRNA also lead to variable mitotic inhibition extending from a short mitotic delay to mitotic catastrophe [44]. While the difficulties in depleting cells completely from cdc25C could in part explain these discrepancies in the requirement for cdc25C for mitosis, an alternative explanation may come from the cell lines used in these studies. The effectiveness of RNA interference for Plk1 was highly dependent on the transformed state of the cell lines used. In highly transformed cells, Plk1 ablation results in cell cycle delay [45] whereas the requirement for Plk1 in non-transformed cells only becomes visible only after clonal selection [44]. From these data, it would appear that cdc25C is an essential component to normal cell mitosis where it likely acts as a checkpoint agent as suggested in prior studies. With increasing levels of transformation, the importance of these checkpoints is reduced as the consequences of aneuploidy is over-ridden by the drive for deregulated growth. We are currently examining if multiple alanine mutant forms of cdc25C disrupt mitosis in more transformed cells.

Finally, show here for the first time that different phospho-forms of human cdc25C exist in mitotic cells, that are not simultaneously phosphorylated on the 3 TP motifs phosphorylated during mitosis in human cells [17]. One potential explaination for these observations is that the binding of the antibody to the phosphorylated residue protects it from threonine phosphatase activity which otherwise dephosphorylates the other sites during the immunoprecipitation process. However, this is highly unlikely to be the case here for at least 2 reasons: Firstly, the immunoprecipitations are performed in the presence of a cocktail of phosphatase inhibitors including 1.0 uM Okadaic acid, tautomycin and calyculin A as well as other phosphatase attenuators such as beta-glycero-phosphate and orthophosphate. Secondly, these observations are backed up by the finding that 2 of the different phospho-forms have spatially separate intracellular localizations during mitosis. Together, these data effectively exclude that a multi TP/SP site amplification loop involving cdk1/cyclin B1 and cdc25C exists in human mitosis. Instead our data suggest the existence of phosphorylation-specific forms of cdc25C with precise cellular localization and function. Our data point to a radically different way in which cdc25C could function in mitosis by targeting specific protein complexes to distinct cellular sites. There is already some previous data in Xenopus that the pT138 form specifically associates with a subunit of phosphatase 2A [28]. It now remains to further determine the nature of the different partner proteins associated with each phospho-site and identify the function of each cdc25C phospho-isoform.

## Materials and Methods

### Cell Culture and Immunofluorescence

Primary immortalized human fibroblasts, T11HT, a generous gift from Dr. Vincent Mouly (Institut de Myologie, Paris) and transformed Hela cells were grown in Dulbeccos MEM, sub-cultured onto glass cover slips and synchronized as described previously [36]. Cells were fixed, extracted and subject to indirect immunofluorescence as described elsewhere [46], [47].

### Antibodies and immunization

Synthetic peptides corresponding to residues 44–52 (T48-DVPRTPVGK and T48-DVPR-*TPVGK (chemically phosphorylated an the central threonine)), 63–71 (T67-LSGGTPKCC and T67-LSGG-*TPKCC) and T130, 127–135 (T130-LCSTPNGLD) and 127–135 (T130-LCS*TPNGLD) of human cdc25C were conjugated with keyhole limpet heamocyanin and injected into rabbits. Peptides were synthesized and coupled by Neosystems, Strasbourg, France. Before immunoblotting, immunoprecipitation or immunofluorescence, sera were double affinity purified on ECH Sepharose 4B columns (Amersham Biosciences, Orsay, France) coupled with the non-phosphorylated or immune phospho-peptides, using the manufacturer's instructions. All antisera where first passed through the non immune- non –phosphorylated peptide column and unbound antibodies subsequently bound and eluted from the phospho-peptide column. Antisera were stored at 4°C, while purified antibodies at −80°C in 5% glycerol.

### Immunoblots, transfection and immunoprecipitation analyses

Total extracts from cells were electrophoretically separated on 10% SDS-polyacrylamide gels and immunoblotted as previously described [36]. Commercial primary antibodies against Plk1 and cdc25C (F8 and H150, Santa Cruz Biotechnology, Santa Cruz, CA), cdc25C (N20, [36] were used at 1∶500. Antibodies to MPM2 were used 1∶500. Affinity purified anti-phospho-cd25C specific antibodies were used at 1∶1000 and anti-tubulin ascites (clone DM1A) at 1∶10000. Mammalian cells were transfected with Lipofectamine 2000 (InVitrogen Inc, Les Ulis, France) according to the manufacturers instructions. All immuno-precipitations were performed as described [36]. Essentially, cells were washed once in ice cold PBS before scraping and collection by centrifugation. Cell pellets were snap frozen at −70°C before lysis in 50 mM Tris-CL (pH 7.5), 150 mM NaCl, 10mM Na-phosphate (pH 7.5), 1.0% (v/v) NP40, 0.5% (v/v) deoxycholate, 0.5% (v/v) Triton X100, 1.0 uM Okadaic acid, 50 uM Tautomycin, 50 uM Calyculin A, 50 mM beta-glycero-phosphate, 10 mM NaF and 2mM Na-Vanadate. This buffer was used throughout except for the final washing of the antibody-protein-A-bead complexes which were washed in PBS (150 mM Na-phosphate, 20 mM K-phosphate, pH 7.5) supplemented with 1.0 uM Okadaic acid, 50 uM Tautomycin, 10 nM Calyculin A, 50 mM beta-glycero-phosphate, 10 mM NaF and 2mM Na-Vanadate.

### Immunofluorescence

For immunofluorescence, cells were fixed in 3.7% (v/v) formalin in PBS or in –20°C methanol as described previously [36]. Anti-phospho-site antibodies were diluted at 1∶2000 for sera or 1∶500 for affinity purified antibodies. Cells were mounted and photographed as described elsewhere [36].

### Site directed mutagenesis

N-terminally HA-tagged human cdc25C (accession number GB:M34065) in pECE was a generous gift from Dr. Franck McKeon, Harvard Medical School. Plasmids were subjected to PCR based site directed mutagenesis using the Quick-change method (Stratagene, La Jolla, USA) according to the manufacturers instructions. Complementary oligonucleotide pairs to:

T48A (5-GTCCAGATGTCCCTAGA**g**CTCCAGTGGGCAAATTT-3),

T67A (5-GCATTTTGTCTGGAGGA**g**CtCCAAAATGTTGCCTCGATC-3),

T130A (5-CACAGCTTCTTTGTAGC**g**CTCCGAATGGTTTGGAC-3) where used.

The open reading frame of each mutant was sequenced entirely. Bacterial expression vectors encoding 6His-cdc25CWt and each T-A mutant were constructed in pET101d using the Topo Challenge system (InVitrogen, Les Ulis, France) according to the manufacturers instructions. Each plasmid was sequenced. Proteins were expressed in in Bl21-DE3 and purified by standard nickel chelating chromatography.

## Supporting Information

Figure S1 Antigenic Peptides and in vitro phosphorylation of cdc25C and mutant forms. Panel A: Multiple sequence alignments of the 3 proline directed threonine phosphorylation sites on human cdc25C phosphorylated at mitosis. Shown are 20 AA sequences at each site in Xenopus, rat, mouse pig and human. Right hand of each block of aligned sequences is the phospho-peptide used as antigen in this study. Panel B: Typical western blot analysis of purified human cdc25C protein using anti-pT48, anti-pT67 and anti-pT130 with (+) or without (−) phosphorylation by cdk1/cyclin B1. Lower panels show the same membranes blotted with anti-cdc25C. Panel C, Purified cdc25C Wt, T48A, T67A and T130A were phosphorylated in vitro and blotted for cdc25C or the anti-phospho-site antibodies. For each protein, the intensity of the bands recognized by the anti-phospho-antibodies was normalized to the staining for cdc25C protein and plotted as a percentage of the overall cdc25C staining. The results were obtained from 3 different experiments.
(0.02 MB PDF)

Figure S2 Staining for cdc25C phospho-T67 cdc25C is specifically abolished by incubation with the phosho-peptide. Asynchronous human fibroblasts were fixed and stained for DNA (panels A, C) or anti-pT67-cdc25C (B and D). Anti-pT67 antibodies were incubated with the non-phosphorylated peptide (B) or phosphorylated peptide (D). Shown are fluorescent photo micrographs of metaphase cells in each panel. Bar 5 µM.
(0.05 MB PDF)

Figure S3 Staining for cdc25C phospho-T67 cdc25C is present on chromatin in transformed cells. Asynchronous U2OS cells were fixed and stained for DNA (panels A, C,E,G) or anti-pT67-cdc25C (B, D, F, H). Shown are fluorescence photo micrographs of representative cells in prophase (A–B), prometaphase (C–D), prometaphase and metaphase (E–F) or anaphase B (G–H). Bar 5 µM.
(0.12 MB PDF)

Figure S4 Staining for cdc25C phospho-T130-cdc25C is specifically abolished by incubation with the phosho-peptide and centrosomal staining is present in transformed cells. Asynchronous human fibroblasts were fixed and stained for DNA (panels A, C) or anti-pT130-cdc25C (B and D). Anti-pT130 antibodies were incubated with the non-phosphorylated peptide (B) or phosphorylated peptide (D). Shown are fluorescence photo micrographs of typical staining patterns in prophase cells in each panel. Panels E and F show staining for pT130-cdc25C in late prometaphase and metaphase U2OS cells. Bar 5 µM.
(0.06 MB PDF)

Figure S5 Endogenous cdc25C proteins are present in the nucleus and on the centrosomes of non-transformed human fibroblasts during mitosis. Asynchronous human fibroblasts were fixed and stained for DNA (panels A, D, G), cdc25C (B, E, H) and tubulin (C,F, I). Shown are fluorescence photo micrographs of cells in early prophase, early prometaphase and late prometaphase. Bar 5 µM.
(0.11 MB PDF)

Figure S6 cdc25C phosphorylated on T130 remains associated with the centrosomes at low temperatures even after Plk1 localization has been lost: Asynchronous human fibroblasts were fixed and stained for DNA (panels A–E), cdc25C-pT130 (F–J) and Plk1 (K–O). Shown are fluorescence photo micrographs of cells in different mitotic phases after incubation at 37°C (A,F,K) or at 4°C for 60 minutes (B,G,L) or 120 minutes (C,H,M; D,I, N; and E,J,O). Bar 5 µM.
(0.05 MB PDF)

Figure S7 Low levels of expression of mutated forms of cdc25C does not perturb the localization of endogenous phospho-cdc25C isoforms. Transformed U2OS cells were transfected with HA-tagged cdc25C T67A or T130A. 24 hours after transfection, cells were fixed and stained for the HA-tagged-cdc25C (panels A and B), cdc25C-pT67 (panel C), cdc25C-pT130 (panel D) or DNA (panels E and F). Shown are typical photo micrographs of cells expressing low levels of HA-tagged cdc25C. Bar 5µm.
(0.07 MB PDF)
