# Supplementary material for: Distinct Pools of cdc25C Are Phosphorylated on Specific TP Sites and Differentially Localized in Human Mitotic Cells
Source: PLoS One. 2010 Jul 26;5(7):e11798. doi: 10.1371/journal.pone.0011798 (PMC2909920; doi:10.1371/journal.pone.0011798)
Supplement: Figure S1 — Antigenic Peptides and in vitro phosphorylation of cdc25C and mutant forms. Panel A: Multiple sequence alignments of the 3 proline directed threonine phosphorylation sites on human cdc25C phosphorylated at mitosis. Shown are 20 AA sequences at each site in Xenopus, rat, mouse pig and human. Right hand of each block of aligned sequences is the phospho-peptide used as antigen in this study. Panel B: Typical western blot analysis of purified human cdc25C protein using anti-pT48, anti-pT67 and anti-pT130 with (+) or without (−) phosphorylation by cdk1/cyclin B1. Lower panels show the same membranes blotted with anti-cdc25C. Panel C, Purified cdc25C Wt, T48A, T67A and T130A were phosphorylated in vitro and blotted for cdc25C or the anti-phospho-site antibodies. For each protein, the intensity of the bands recognized by the anti-phospho-antibodies was normalized to the staining for cdc25C protein and plotted as a percentage of the overall cdc25C staining. The results were obtained from 3 different experiments. (0.02 MB PDF) [file pone.0011798.s001.pdf]

A

```
T48:
Xl_cdc25B  SPEQPLTPVT D.LAVGFSNL                     pT48
Xl_cdc25C  SPEQPLTPVT D.LAVGFSNL
Hs_cdc25C  CPDVPRTPV. GKFLGDSANL           Peptide: DVPRpTPVGK
Sc_cdc25C  SSDRPGTPLK KKLFGDSANL
Mm_cdc25C  RSDFPESP.K DKLH.DSANL
Mm_cdc25B  EPTASSSPVT T.LTQTMHNL
Rn_cdc25B  ERAASSSPVT T.LTQTMYNL
Hs_cdc25B  VRAAASSPVT T.LTQTMHDL
Mm_cdc25A  .AAGGLSPVT N.LTVTMDQL
Rn_cdc25A  .RAGGLSPVT N.LTVTMDQL
Hs_cdc25A  .AAGGLSPVT N.LTVTMDQL
Xl_cdc25A  .ADTALSPVT S.LALNMDQL

T67:
Xl_cdc25B STFSGETPK. .......... .RCLDLSNL.          pT67
Xl_cdc25C STFSGETPK. .......... .RCLDLSNL.
Hs_cdc25C SILSGGTPK. .......... .CCLDLSNLS  Peptide:LSGGpTPKCC
Sc_cdc25C SILSGGTPK. .......... .RCLDLSNLS
Mm_cdc25C SILSGGTPK. .......... .CCLDLSNLS
Rn_cdc25C ..LSGGTPK. .......... .CCLDLS..
Mm_cdc25B AGLGSEPPKA QVGSLSFQNR LADLSLSR..
Rn_cdc25B AGLGSETPKT QVGSLSFQNR LTDLSLSR..
Hs_cdc25B AGLGS..... .......RSR LTHLSLSR..
Mm_cdc25A EGLGSD.... ......CEK. ...MEV.R..
Rn_cdc25A EGLGSD.... ......YEKP ...MDV.R..
Hs_cdc25A QGLGSD.... ......YEQP ...LEV.K..
Xl_cdc25A AGLGSQ.... ......CETP TRKLED.R..

T130:
Xl_cdc25B  NMNSVLPHLL CSTP.SFKKA                    pT130
Xl_cdc25C  NMNSVLPRLL CSTP.SFKKT
Hs_cdc25C  .MKCSPAQLL CSTPNGLDR.           Peptide:LCSpTPNGLD
Sc_cdc25C  .LKCSPAQLL CSTPNALDH.
Mm_cdc25C  .MKGIPVQLL CSTPNAMNH.
Rn_cdc25C  ....IPAQLL CSTPNALDH
Mm_cdc25B  RFRSLPVRLL EHSP.VL.QS
Rn_cdc25B  VFASEAA... GHSP.VL.QN
Hs_cdc25B  RFQSMPVRLL GHSP.VL.RN
Mm_cdc25A  RINSLPQKLL GCSP.ALKRS
Rn_cdc25A  RINCLPQKLL GCSP.ALKRS
Hs_cdc25A  RIHSLPQKLL GCSP.ALKRS
Xl_cdc25A  RRNSLPQNLL GSSP.AFKRN

S214:
Xl_cdc25B  RLYRSPSMPE KLD....RPM                    pS214
Xl_cdc25C  RLYRSPSMPE KLD....RPM
Hs_cdc25C  GLYRSPSMPE NLN....RPR
Sc_cdc25C  CLYRSFSLPD SLN....SPG
Mm_cdc25C  .......... ..........           Peptide:GLYRpSPSMP
Mm_cdc25B  RLFRSPSMPC SV....IRPI
Rn_cdc25B  RLFRSPSMPC SV....IRPI
Hs_cdc25B  RLFRSPSMPC SV....IRPI
Mm_cdc25A  .LFDSPSPCG S....STRAV
Rn_cdc25A  .LFDSPSPCS STSSCSTRAV
Hs_cdc25A  .LFDSPSLCS S....STRSV
Xl_cdc25A  .....PN... ....CTTKPA
```

B

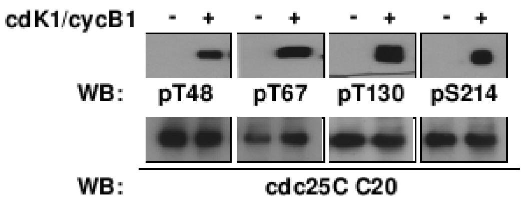

C

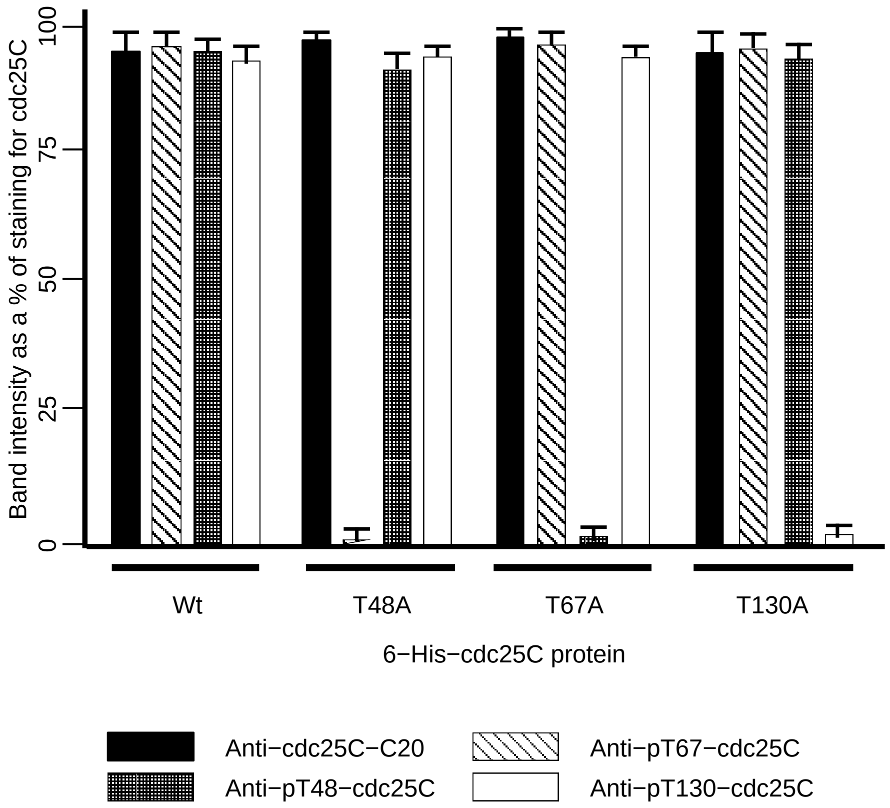

Franckhauser et al , Supplemental Figure 1.
