# Supplementary figures and images for: Distinct Pools of cdc25C Are Phosphorylated on Specific TP Sites and Differentially Localized in Human Mitotic Cells
Source: PLoS One. 2010 Jul 26;5(7):e11798. doi: 10.1371/journal.pone.0011798 (PMC2909920; doi:10.1371/journal.pone.0011798)

DNA

anti-pT67-cdc25C

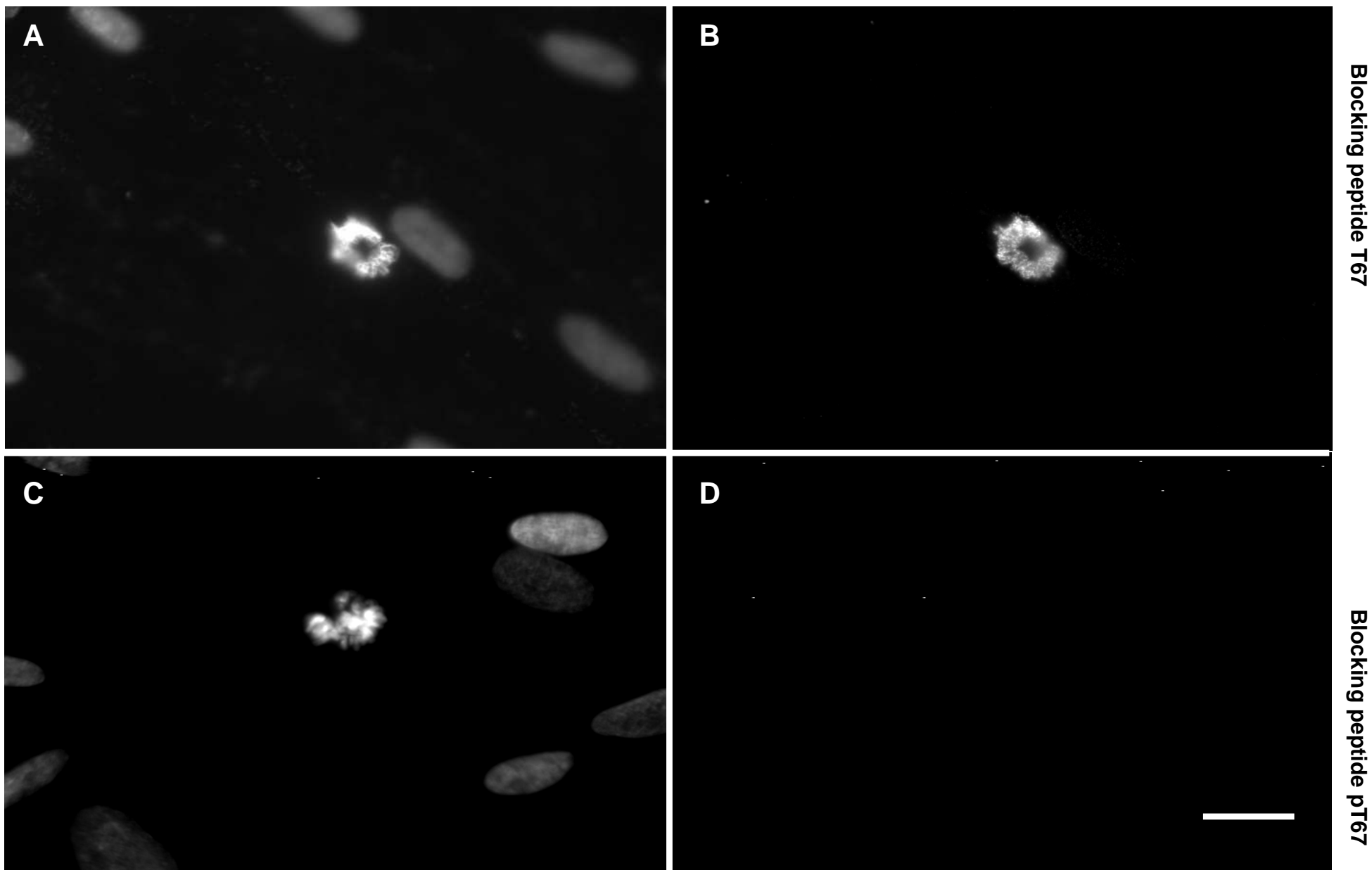

Supplement: Figure S2 — Staining for cdc25C phospho-T67 cdc25C is specifically abolished by incubation with the phosho-peptide. Asynchronous human fibroblasts were fixed and stained for DNA (panels A, C) or anti-pT67-cdc25C (B and D). Anti-pT67 antibodies were incubated with the non-phosphorylated peptide (B) or phosphorylated peptide (D). Shown are fluorescent photo micrographs of metaphase cells in each panel. Bar 5 µM. (0.05 MB PDF) [file pone.0011798.s002.pdf]

**DNA**

**anti-pT67**

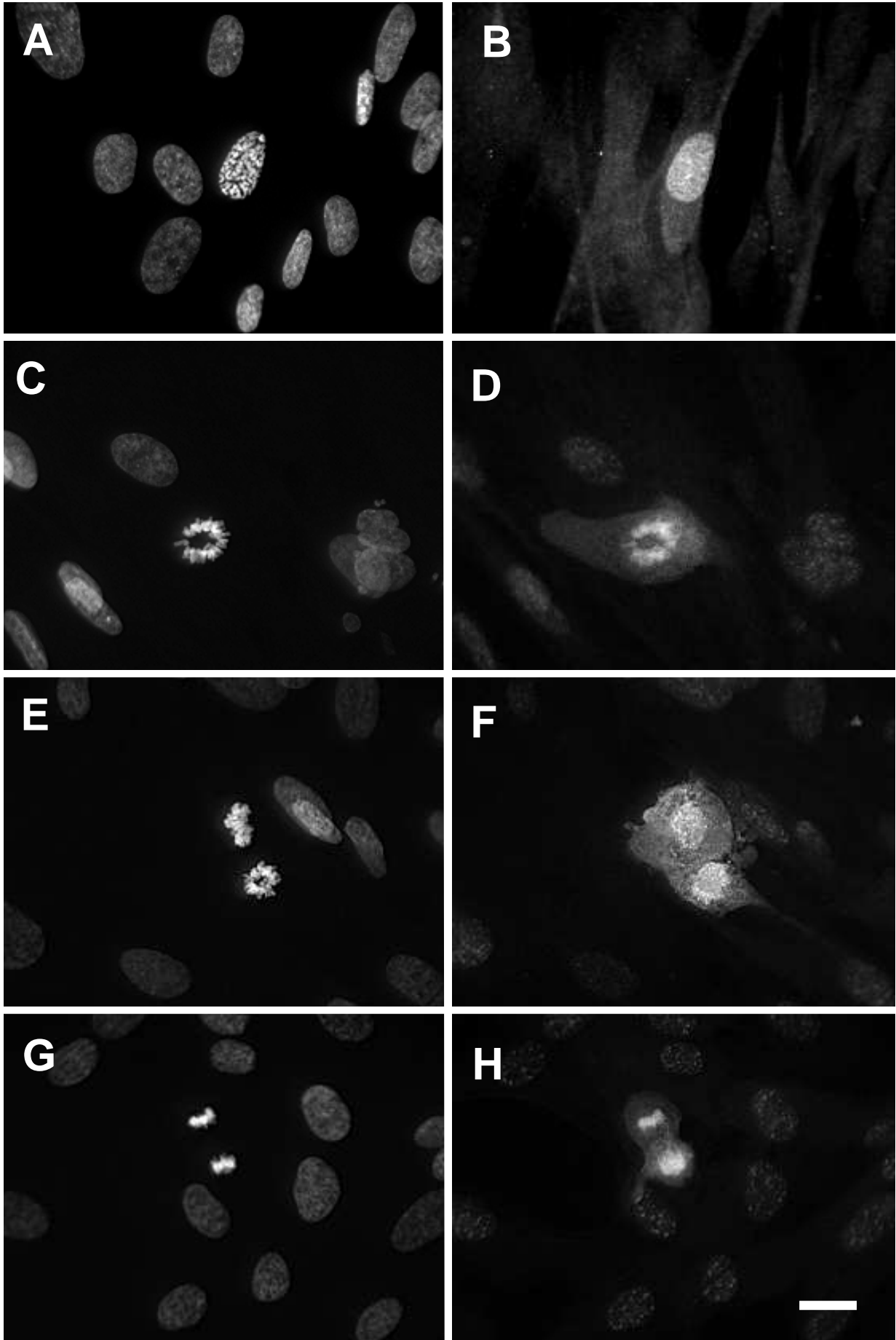

Supplement: Figure S3 — Staining for cdc25C phospho-T67 cdc25C is present on chromatin in transformed cells. Asynchronous U2OS cells were fixed and stained for DNA (panels A, C,E,G) or anti-pT67-cdc25C (B, D, F, H). Shown are fluorescence photo micrographs of representative cells in prophase (A–B), prometaphase (C–D), prometaphase and metaphase (E–F) or anaphase B (G–H). Bar 5 µM. (0.12 MB PDF) [file pone.0011798.s003.pdf]

Human diploid fibroblasts

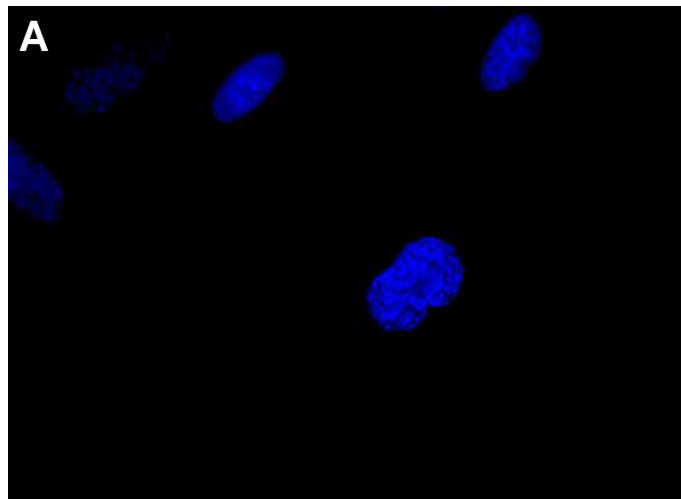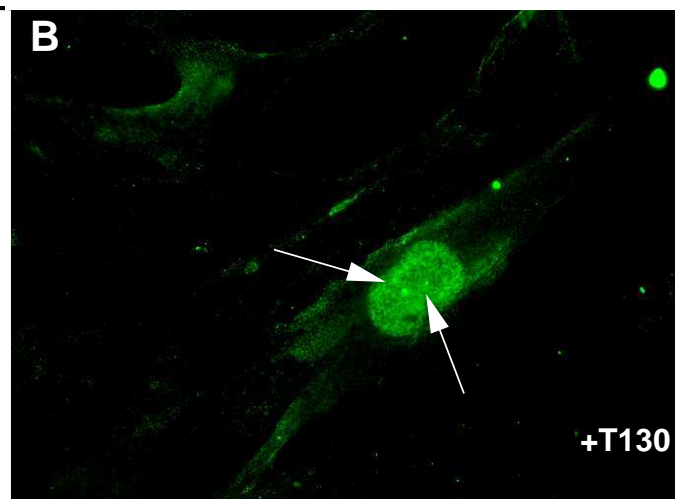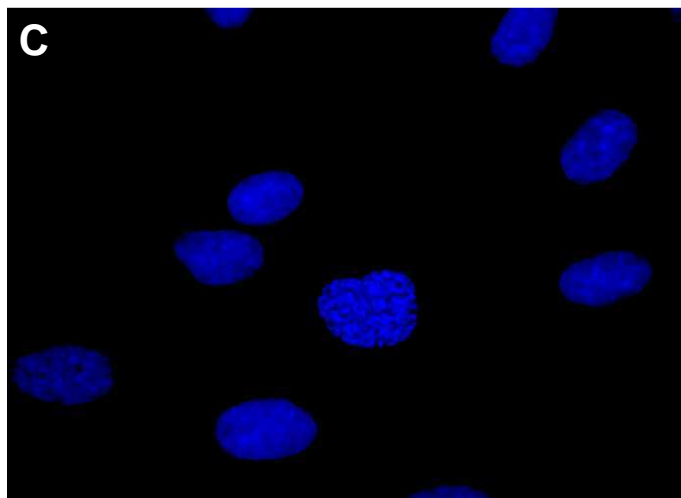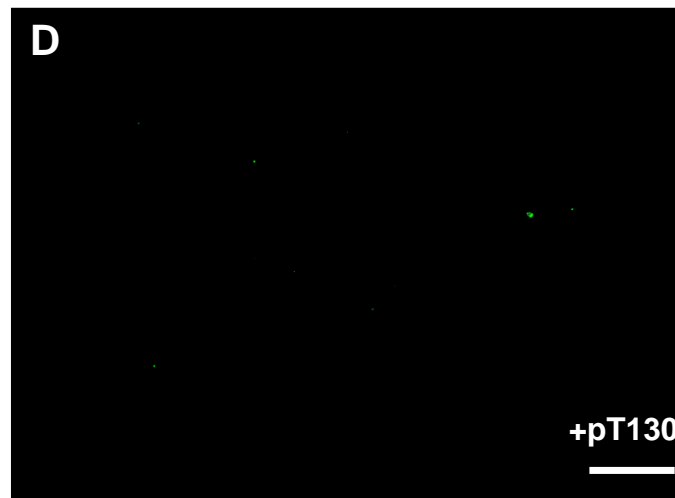

U2OS

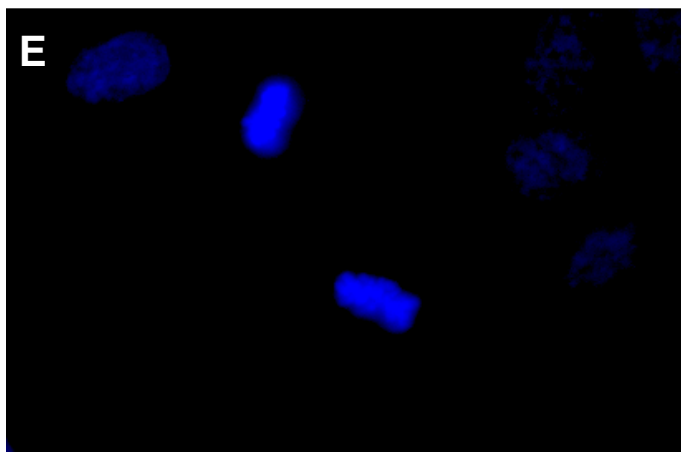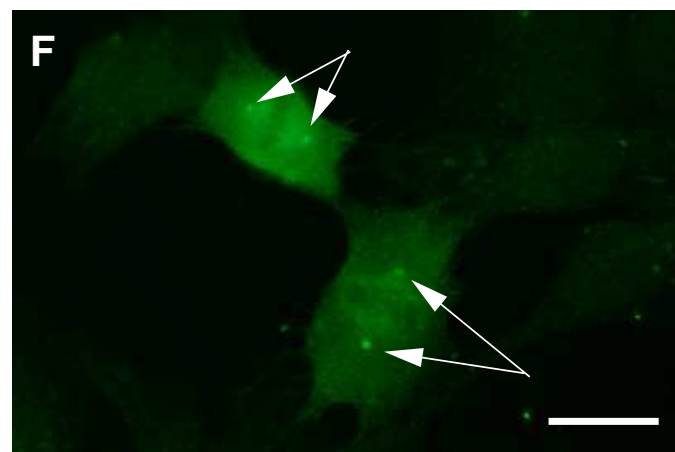

Supplement: Figure S4 — Staining for cdc25C phospho-T130-cdc25C is specifically abolished by incubation with the phosho-peptide and centrosomal staining is present in transformed cells. Asynchronous human fibroblasts were fixed and stained for DNA (panels A, C) or anti-pT130-cdc25C (B and D). Anti-pT130 antibodies were incubated with the non-phosphorylated peptide (B) or phosphorylated peptide (D). Shown are fluorescence photo micrographs of typical staining patterns in prophase cells in each panel. Panels E and F show staining for pT130-cdc25C in late prometaphase and metaphase U2OS cells. Bar 5 µM. (0.06 MB PDF) [file pone.0011798.s004.pdf]

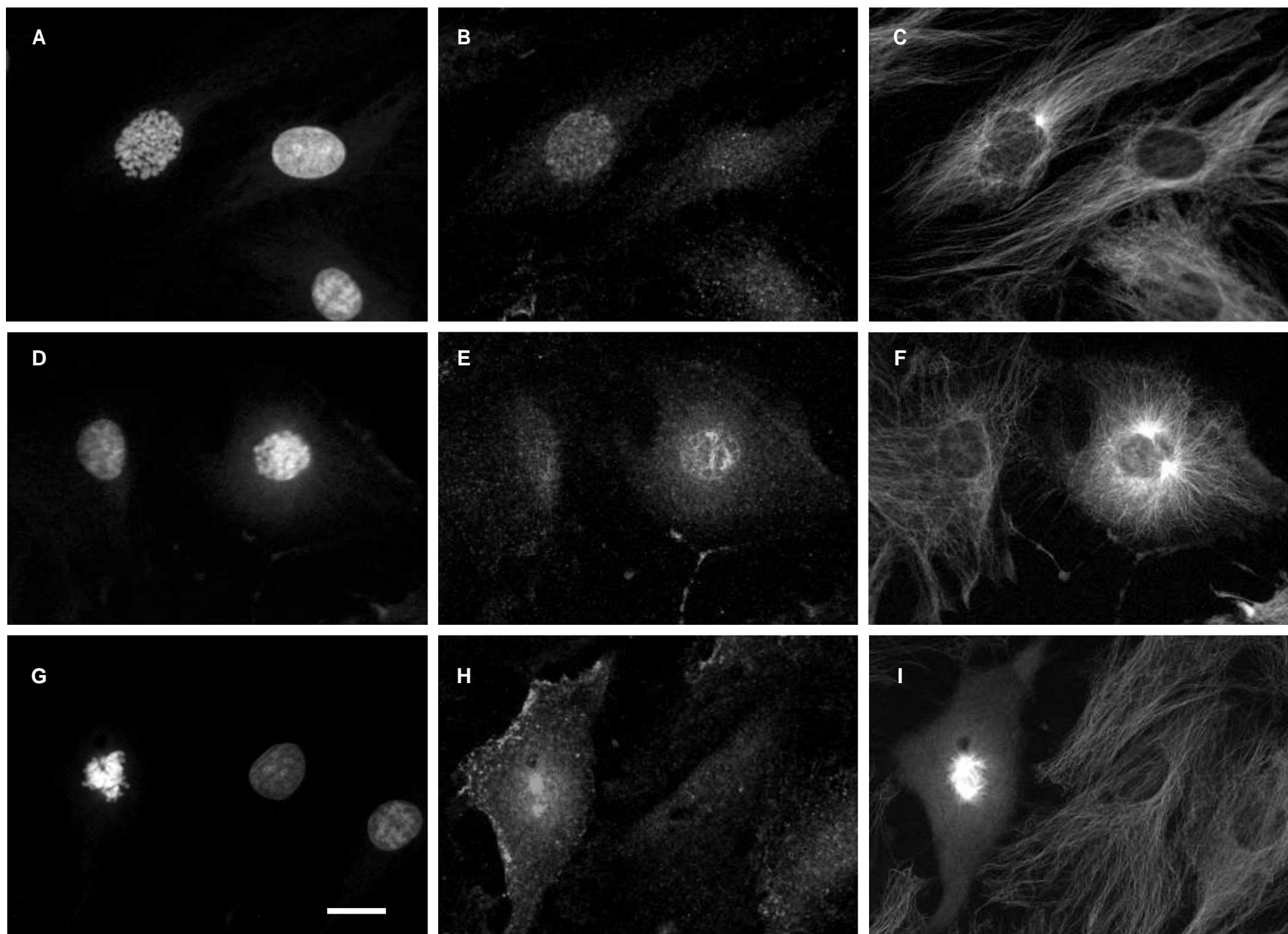

Supplement: Figure S5 — Endogenous cdc25C proteins are present in the nucleus and on the centrosomes of non-transformed human fibroblasts during mitosis. Asynchronous human fibroblasts were fixed and stained for DNA (panels A, D, G), cdc25C (B, E, H) and tubulin (C,F, I). Shown are fluorescence photo micrographs of cells in early prophase, early prometaphase and late prometaphase. Bar 5 µM. (0.11 MB PDF) [file pone.0011798.s005.pdf]

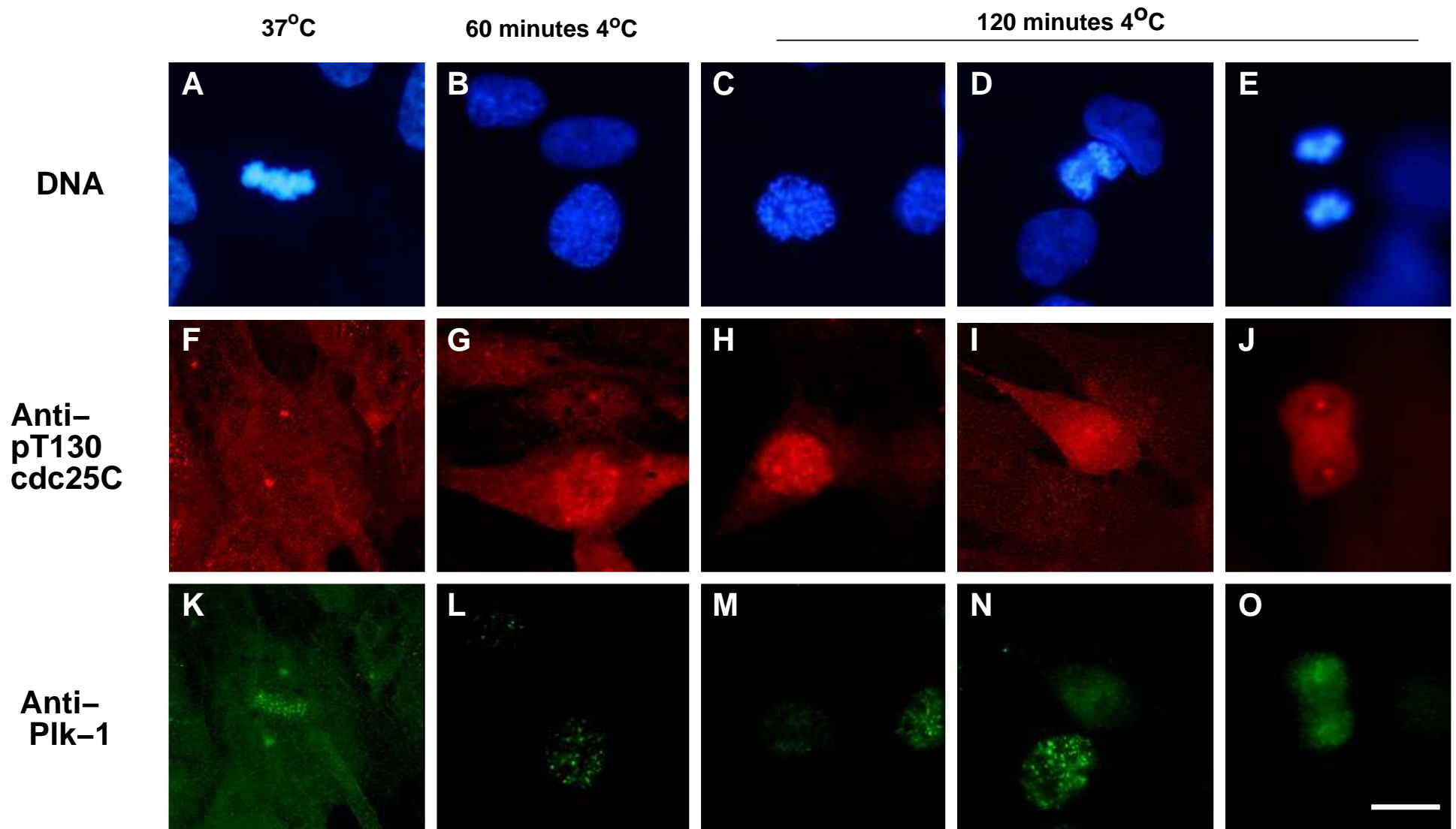

Supplement: Figure S6 — cdc25C phosphorylated on T130 remains associated with the centrosomes at low temperatures even after Plk1 localization has been lost: Asynchronous human fibroblasts were fixed and stained for DNA (panels A–E), cdc25C-pT130 (F–J) and Plk1 (K–O). Shown are fluorescence photo micrographs of cells in different mitotic phases after incubation at 37°C (A,F,K) or at 4°C for 60 minutes (B,G,L) or 120 minutes (C,H,M; D,I, N; and E,J,O). Bar 5 µM. (0.05 MB PDF) [file pone.0011798.s006.pdf]

HA-T67A-cdc25C mutant

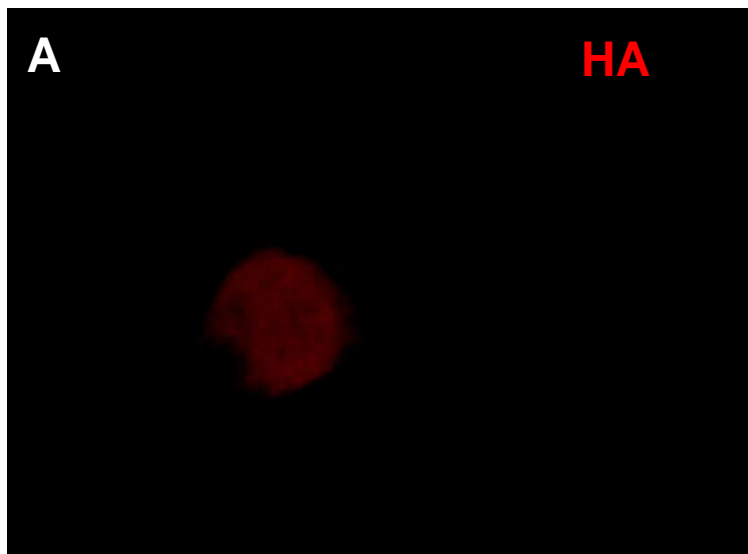

HA-T130A-cdc25C mutant

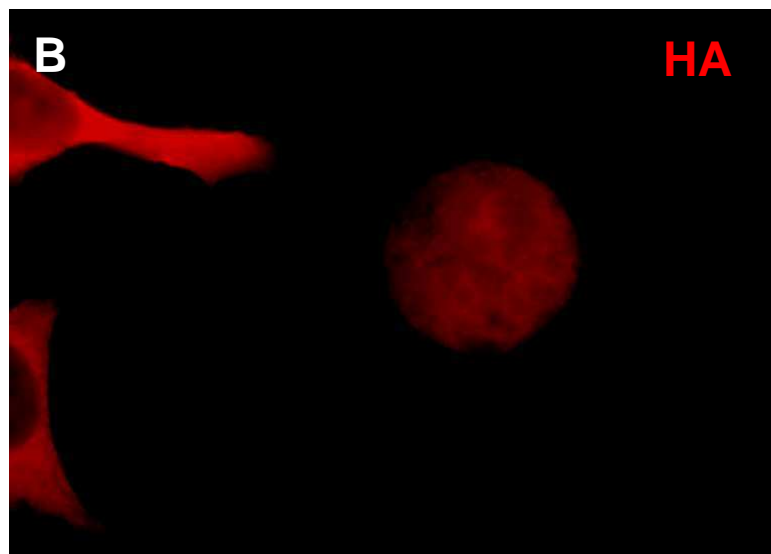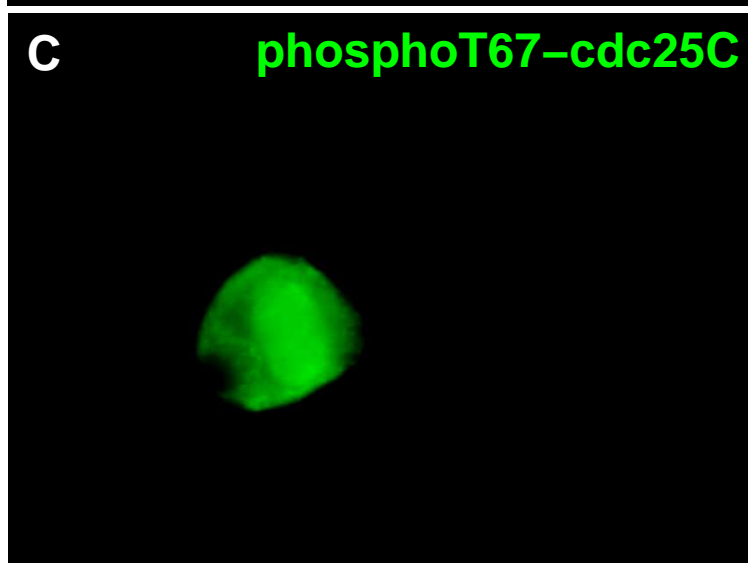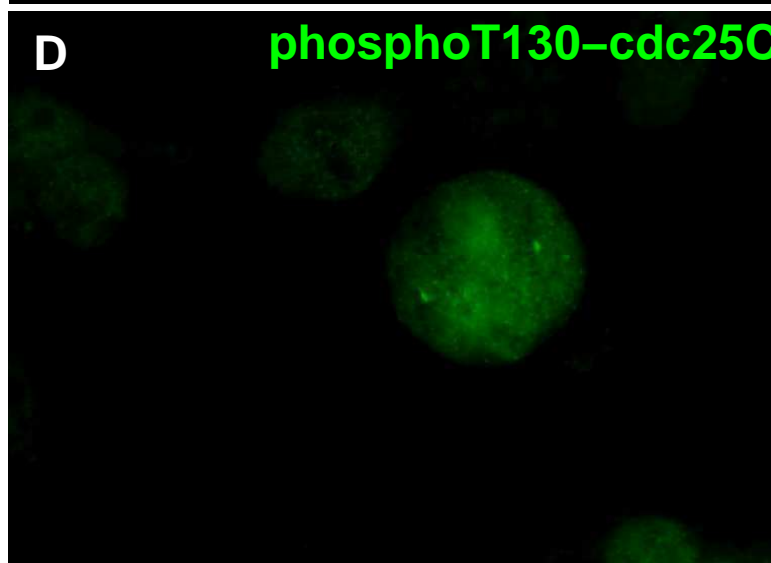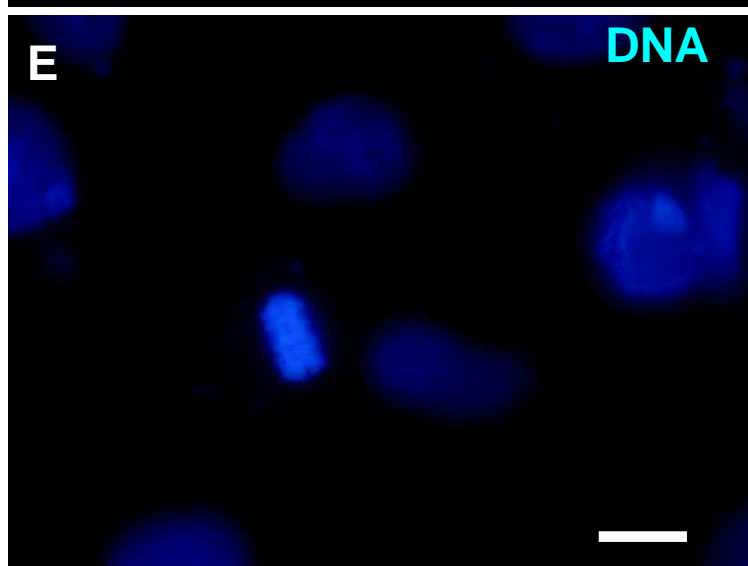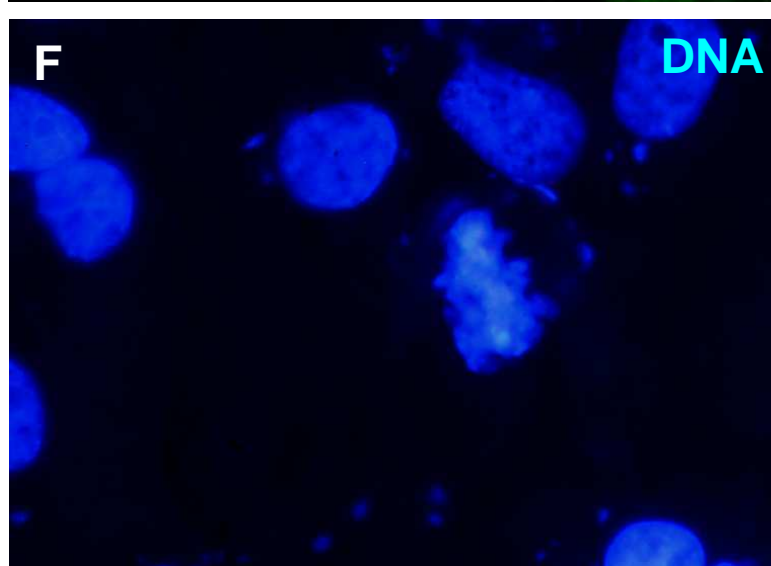

Supplement: Figure S7 — Low levels of expression of mutated forms of cdc25C does not perturb the localization of endogenous phospho-cdc25C isoforms. Transformed U2OS cells were transfected with HA-tagged cdc25C T67A or T130A. 24 hours after transfection, cells were fixed and stained for the HA-tagged-cdc25C (panels A and B), cdc25C-pT67 (panel C), cdc25C-pT130 (panel D) or DNA (panels E and F). Shown are typical photo micrographs of cells expressing low levels of HA-tagged cdc25C. Bar 5µm. (0.07 MB PDF) [file pone.0011798.s007.pdf]
